# *R132H IDH1* sensitizes glioma to the antiproliferative and cytotoxic effects of BET inhibition

**DOI:** 10.1007/s00432-022-04018-w

**Published:** 2022-04-25

**Authors:** Thomas K. Sears, Kevin D. Woolard

**Affiliations:** 1 Department of Neurological Surgery, Northwestern University, Chicago, IL USA; 2 Department of Pathology, Microbiology, and Immunology, UC Davis School of Veterinary Medicine, Davis, CA USA

**Keywords:** Mutant isocitrate dehydrogenase 1/2 (*IDH1/2*), Glioma, Bromodomain inhibition, Bromodomain and extraterminal (BET), JQ1

## Abstract

**Introduction:**

Mutations in *isocitrate dehydrogenase 1/2* (IDH^mut^) identify a subset of gliomas that exhibit epigenetic dysregulation via aberrant DNA methylation. These tumors are ultimately fatal and lack effective therapeutic strategies. Considering the epigenetic dysregulation of IDH^mut^ gliomas, we hypothesized that epigenetic-targeting drugs may yield therapeutic benefits in gliomas bearing IDH^mut^. One set of targets includes the bromodomain and extraterminal (BET) family of transcriptional coactivators.

**Methods:**

We used TCGA data from glioma patients to determine whether BET proteins affect patient survival differently based on IDH status. Follow-up experiments using a set of IDH wildtype/mutant glioma cultures, as well as an IDH wildtype glioblastoma cell line expressing exogenous *R132H IDH1*, focused on cell health assays to investigate whether IDH^mut^ was associated with increased sensitivity to the BET inhibitor JQ1. Immunoblots were used to evaluate the molecular response to JQ1 in these cultures.

**Results:**

We identified that high BRD4 expression associated with decreased survival only in IDH^mut^ glioma patients. Cell viability analysis showed that IDH^mut^ sensitized glioma cells to delayed cytotoxicity (10 days) in response to JQ1. Early effects of JQ1 (3 days) were primarily antiproliferative, with IDH^mut^ glioma exhibiting a modest increase in sensitivity. Finally, exogenous *R132H IDH1* expression in a resistant IDH wildtype cell line recapitulated the JQ1-mediated delayed cytotoxicity seen in our endogenous IDH^mut^ glioma cells.

**Conclusion:**

Overall, these data suggest that BRD4 enhances malignancy primarily in gliomas bearing IDH^mut^ and is associated with greater sensitivity to BET inhibition. The finding that BET inhibition primarily exhibits delayed cytotoxicity may be overlooked in conventional short endpoint dose–response assays. Follow-up mechanistic and animal studies will help address the translational potential of these findings.

**Supplementary Information:**

The online version contains supplementary material available at 10.1007/s00432-022-04018-w.

## Introduction

Mutations in *isocitrate dehydrogenase 1/2* (IDH^mut^) occurs early in gliomagenesis (Sanson et al. 2009) and lead to neomorphic production of the oncometabolite D-2-hydroxyglutaraste (D2HG). High intracellular levels of D2HG result in inhibition of DNA and histone demethylase enzymes, thereby leading to epigenetic dysregulation through increased DNA and histone methylation. DNA and histone methylation is associated with epigenetic repression of genes through formation of heterochromatin. Of interest is the stability of this DNA methylation observed in IDH^mut^ glioma tumors, with much of the CpG island methylation that defines IDH^mut^ glioma being retained in patients post-resection and chemotherapy (Mazor et al. 2017). Based on this, we became interested in investigating epigenetic inhibitors as potential therapeutic targets for IDH^mut^ glioma.

The bromodomain and extraterminal (BET) family of proteins represents a class of epigenetic “readers” that bind to acetylated chromatin residues to facilitate transcriptional activation of nearby genes (Jain et al. 2017), hence these proteins are also termed transcriptional coactivators. These BET proteins have proven roles in various physiological and pathophysiological processes such as maintenance of stem-cell populations (Horne et al. 2015; Wu et al. 2015; Bolden et al. 2014) and promotion of tumorigenesis (Xu et al. 2018). The BET family of proteins included BRD2, BRD3, BRD4, and the testis-specific isoform BRDT (Taniguchi 2016). Most research has focused on the function of the BRD4 isoform for its major role in transcriptional pause-release via recruitment of the elongation factor complex PTEF-b to transcriptionally paused genes (Miller et al. 2017). With the observation that BET proteins are upregulated in cancer (Pastori et al. 2014) and facilitate transcription of oncogenes such as c-MYC (Delmore et al. 2011), this led to the concept that BET proteins, particularly BRD4, facilitate tumorigenesis via transcriptional coactivation of oncogenes. Inhibition of receptor tyrosine kinase (RTK) pathways, such as PDGFR signaling (Wang et al. 2015), has also been observed via BET inhibition. These observations support the investigation of small molecule BET inhibitors for utilization as potential cancer therapeutics.

In the 1990s, Mitsubishi Pharmaceuticals identified the first known bromodomain inhibitors using the benzodiazepine pharmacophore thienodiazepam. This chemical scaffold was eventually further improved upon as a lead compound by Filippakopoulos et al. in 2011 with successful development of the selective bromodomain inhibitor JQ1 (Filippakopoulos et al. 2010). This drug exhibited pan-BET inhibition activity, with similar binding affinity for BRD2/3/4 but low binding to BRDT. JQ1 exhibited very promising pharmacodynamic properties when investigated as an anticancer therapeutic by targeting oncogene expression, such as c-MYC. This drug was also shown to readily penetrate the blood–brain barrier, but the pharmacokinetics proved troublesome with a plasma half-life of just 1–1.5 h (Filippakopoulos et al. 2010). Nevertheless, this compound has been used extensively in the literature as a tool compound to investigate BET-related functions in various ailments such as cancer (Cheng et al. 2013) and inflammatory diseases (Belkina et al. 2013).

Of interest is the role of MYC proteins in the progression and malignancy of IDH^mut^ gliomas, with MYC activation associated with progression from low grade glioma to high-grade GBM (Korshunov et al. 2019). Additionally, it has been shown that other IDH^mut^ cancers, such as cholangiocarcinomas and colorectal cancers, have also exhibited enhanced sensitivity to BET inhibitors (Fujiwara et al. 2018; McCleland et al. 2016). Therefore, we became interested in whether IDH^mut^ gliomas more heavily rely upon the oncogenic functions of BET transcriptional coactivators for cellular survival and proliferation.

Here, we investigate the role of BET protein function in IDH wildtype (IDH^wt^) vs IDH^mut^ glioma using The Cancer Genome Atlas (TCGA) clinical data and in vitro cell assays. We utilized a panel of IDH^wt/mut^ glioma cell lines, representing Grade 3 and Grade 4 gliomas, along with an IDH^wt^ cell line bearing a stable transgenic *R132H IDH1* mutation to investigate the hypothesis that IDH^mut^ gliomas are more sensitive to the antiproliferative and/or cytotoxic effects of BET inhibition.

## Materials and methods

### Glioma cell culture

0827, 0923, and 0905 GBM stem-like cells were previously isolated by Dr. Howard Fine’s lab and have been previously characterized for GBM stem-like properties and genomic alterations (Toledo et al. 2015; Ene et al. 2012; Son et al. 2009). BT142 (Luchman et al. 2011) is a grade III anaplastic astrocytoma and was purchased from ATCC, while TB096 (Moure et al. 2019), another grade III anaplastic astrocytoma, was a gift from Dr. Hai Yan at Duke University. Validation of *IDH1* mutations and 2-HG production has been previously conducted by our lab for all cell lines used in this study (Sears et al. 2021). All cell lines were grown in defined neurobasal cell culture medium that includes B-27 and N2 supplements along with EGF and FGF growth factors (NBE). 0905 and BT142 required 100 ng/mL PDGF-AB in the medium, while TB096 required culture conditions containing half NBE and half DMEM with 10% FBS. All cell cultures were cultured at 37C and 5% CO_2_, included penicillin–streptomycin, and frequently monitored for mycoplasma contamination. Additionally, all cells were cultured in suspension as neurospheres except for TB096 which exhibited adherent growth. Cell culture medium was replenished 3 ×/week. JQ1 (Millipore Sigma; SML1524) was dissolved in 100% ethanol, sterile filtered, and diluted to 1000 × in sterile ethanol for drug treatments. Vehicle concentration was 0.1% or less for all treatments.

### TCGA survival analysis

The UCSC Xena platform (Goldman et al. 2020) was utilized for analysis of patient overall survival in IDH^wt/mut^ glioma based on BET isoform (*BRD2/3/4*) gene expression. Low grade glioma and glioblastoma patients were analyzed together via the TCGA GBMLGG cohort dataset. IDH^mut^ astroctyomas and oligodendrogliomas were distinguished based on 1p/19q codeletion status. This dataset includes information on copy number alterations, DNA methylation, gene expression, somatic mutations, and survival for each patient.

### Cell viability and apoptosis assays

A Luminex Guava Muse Cell Analyzer was utilized to conduct cell viability and apoptosis assays using a Muse Cell Count and Viability Kit or a Muse Annexin V and Dead Cell Kit. Cells prepared as single-cell suspensions were analyzed on the Muse Cell Analyzer according to the manufacturer’s protocols. Briefly, cells were trypsinized (0.05%), mixed with Cell Count and Viability reagent or 1:1 NBE to Annexin V and Dead Cell reagent, and then incubated according to the manufacturer’s protocol. Stained samples were then run on the Muse Cell Analyzer, and gates were set based on a positive control sample.

### Immunoblotting

Cell culture samples were washed with 1 × DPBS, pelleted via centrifugation, flash-frozen in liquid nitrogen, and stored at − 80 °C for later cell lysis. For preparation of whole cell lysates, cell pellets were resuspended in 1 × RIPA buffer supplemented with 1 × Halt protease and phosphatase inhibitor and then pellets to homogenized via a probe sonicator for 3 × 20 s at 30% amplitude. Insoluble material was pelleted via centrifugation, and the supernatant protein concentration quantified via Bradford assay. SDS-PAGE and transfer steps were conducted using the Novex immunoblot system. All blots used NuPage 4–12% Bis–Tris gradient gels. Blots were visualized via a ProteinSimple Fluorchem E Imager. Antibody information can be found in Table S1.

### BrdU incorporation assays

BrdU incorporation assays were conducted using a BD Pharmingen BrdU Flow kit according to the manufacturer’s protocol. Briefly, cells were treated for 48 h with drug and then pulsed with 10 µM BrdU, except for our background stain control, for 2.5 h before washing, fixing, and storing at − 80 °C in solution of 1:9 FBS to DMSO. For analysis on flow cytometer, cells were thawed, permeabilized, and incubated with anti-BrdU antibody according to manufacturer’s protocol. Stained cell samples and unstained controls were run on a Beckman Coulter Cytomics FC500 flow cytometer with FITC filter applied. Forward- and side scatter voltages and gates were set using the No BrdU control, whereas FITC voltages were set using a BrdU positive control sample to determine BrdU + and BrdU − cells.

### Plasmids

pSLIK-IDH1-FLAG (Addgene plasmid # 66802; http://n2t.net/addgene:66802; RRID: Addgene_66802) and pSLIK-GFP (Addgene plasmid # 66844; http://n2t.net/addgene:66844; RRID: Addgene_66844) were gifts from Christian Metallo37. These plasmids were provided as bacterial stabs and once acquired were grown in LB and clones screened for fidelity via restriction digestion and Sanger sequencing. Acceptable clones were then further grown in LB and plasmids isolated via Qiagen Maxiprep kit. Plasmid concentration was then quantified prior to development of lentivirus.

### Lentivirus production

Lentiviral vectors were generated by transfecting either pSLIK-GFP or pSLIK-IDH1-R132H-FLAG transfer plasmids with an envelope plasmid (pCMV-VSV-G) and packaging plasmid (pCMV-dR8.2 dvpr). These three plasmids were transfected into 293 T cells using Polyplus Jetprime transfection reagent according to the manufacturer’s protocol and then cell culture media was collected every 24 h for 3 days and stored at − 80 °C. Lentivirus-containing cell culture medium was centrifuged on tabletop centrifuge to remove cell debris, and then further centrifuged on a Beckman Coulter Optima XL-100 K Ultracentrifuge at 4C and 25,000 g for 1 h. Lentivirus-containing pellets were resuspended in 1 × DPBS and then assayed via Takara Bio’s Lenti-X GoStix for presence of adequate lentiviral titer.

### Lentiviral transduction of cell lines

0923 GBM cells were plated in 6-well plates and then lentivirus was titrated over 5 wells. After 2 days inoculation, lentivirus-containing NBE was swapped out with hygromycin-containing (150 μg/mL) NBE. After 2 weeks in hygromycin, transduced cells were grown in normal NBE and then validated for *R132H IDH1* gene expression via immunoblot and 2HG production via ELISA.

### Statistical analysis

2-way ANOVA and Student’s *t* test statistical analyses were performed via GraphPad Prism 8.0 software package. Logrank statistical analysis of Kaplan–Meier survival curves was conducted via the UCSC Xena Browser platform. All replicate analyses are presented as mean ± standard deviation of the mean for three independent replicates at a significance level (α) of 0.05 unless otherwise indicated in the figure legends. Statistical significance was denoted in figures using the following system: *p ≤ 0.05; **p ≤ 0.001; ***p ≤ 0.0001.

## Results

### TCGA data shows differential roles for BET isoforms in glioma based on *IDH* status

**Fig. 1:**
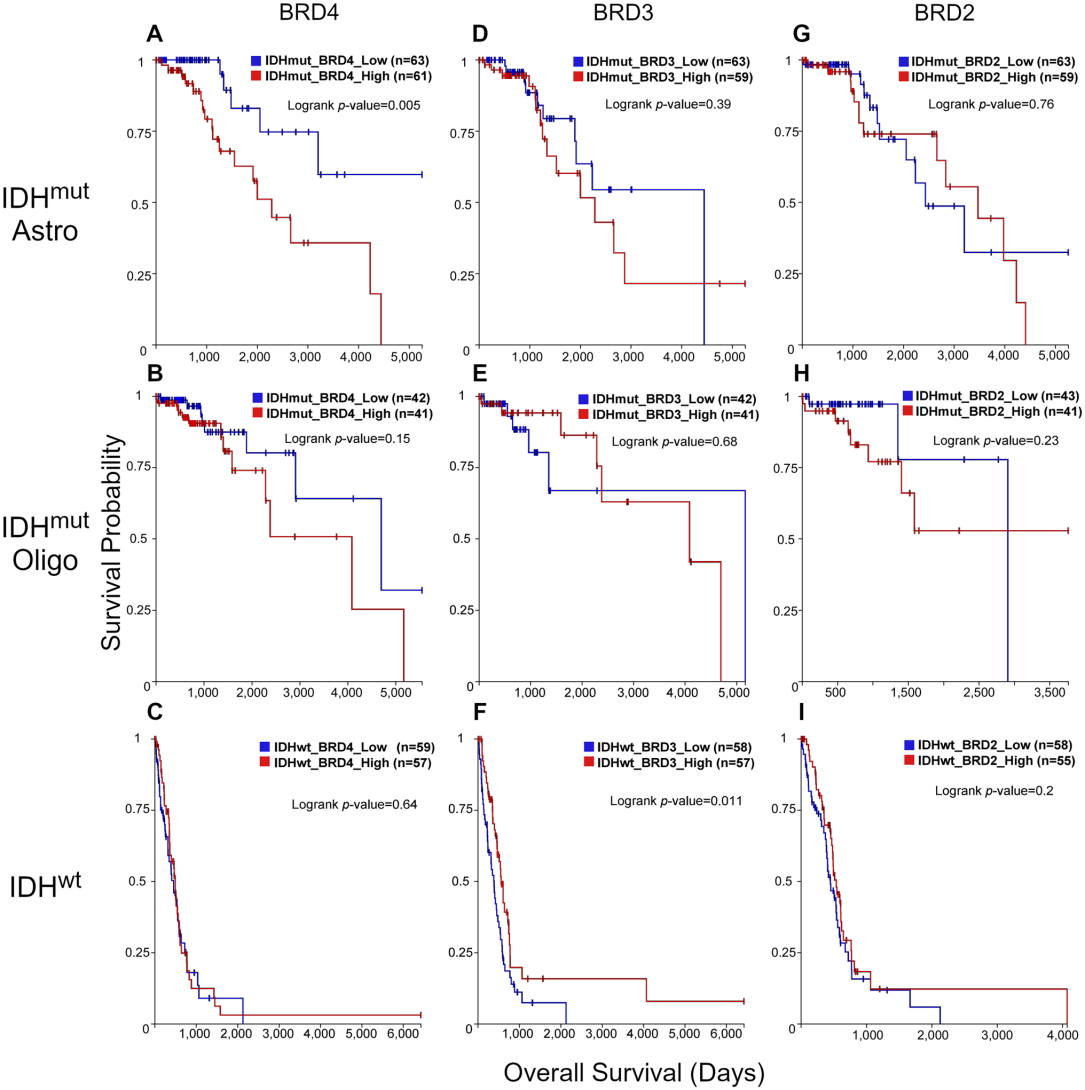
Survival analysis reveals differential reliance on BET isoform between IDH WT and mutant glioma. **A**–**I** Analysis of overall survival in IDH^wt/mut^ low grade glioma and glioblastoma patients stratified based on BRD2, BRD3, and BRD4 expression. IDH^mut^ astrocytomas and oligodendrogliomas were distinguished based on 1p/19q codeletion status. Data was acquired via the UCSC Xena browser using the TCGA GBMLGG cohort. Statistical analyses were performed using the logrank test with sample size and *p* values presented in each figure panel

We first delved into TCGA data via the UCSC Xena browser using the low-grade glioma and glioblastoma (GBMLGG) cohort dataset. GBMLGG data was segregated based on *IDH1/2* status, with IDHmut gliomas further stratified between astrocytomas (1p/19q intact) and oligodendrogliomas (1p-19q codeleted), and overall survival plotted based on median BET isoform (*BRD2/3/4*) expression levels. We found that enhanced *BRD4* expression was associated with a significant decrease in overall survival only in IDH^mut^ astrocytoma patients (Fig. 1A). A similar, yet not significant (*p* = 0.15), trend was observed in IDH^mut^ oligodendrogliomas (Fig. 1B). This decrease in survival with high *BRD4* expression was not seen in IDH^wt^ patients (Fig. 1C). In contrast to the *BRD4* TCGA data, we found that *BRD3* expression only influenced IDH^wt^ patient survival, with high *BRD3* expression associated with a significant increase in overall survival (Fig. 1D–F). *BRD2* expression levels did not correlate with a significant increase or decrease in overall survival in either IDH^wt^ or IDH^mut^ glioma patients (Fig. 1G–I). Overall, these data suggest that increased *BRD4* activity results in enhanced malignancy only in IDH^mut^ glioma, particularly astrocytomas, whereas increased *BRD3* activity results in enhanced survival only in IDH^wt^ glioma. Further, this suggests that IDH^mut^ glioma would be particularly sensitive to BET inhibition and supports investigation of BET inhibitors as a therapeutic strategy for the treatment of IDH^mut^ glioma.

### Bromodomain inhibition by JQ1 elicits delayed cytotoxicity only in IDH^mut^ glioma

**Fig. 2:**
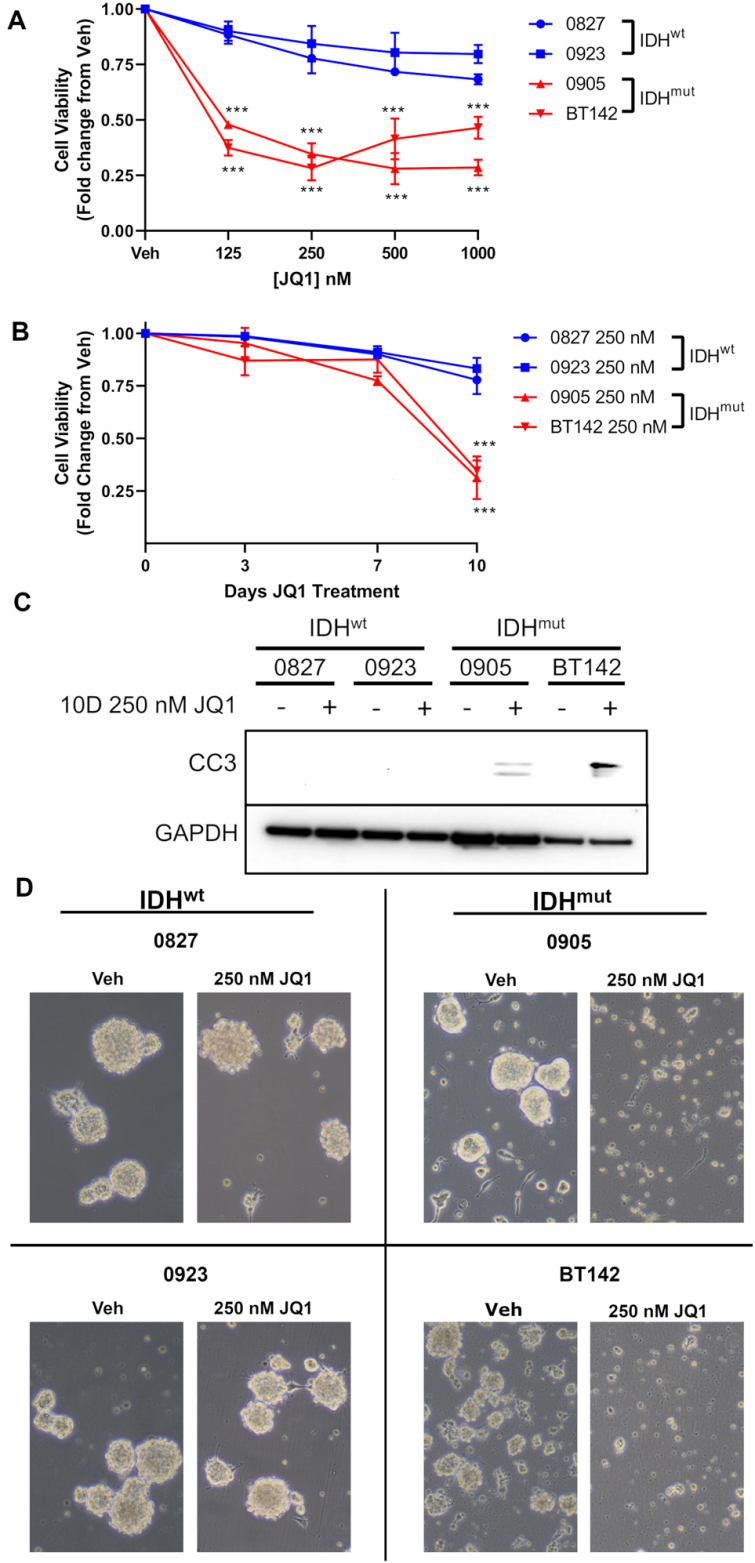
A preferential cytotoxic response is observed in IDH^mut^ glioma cells, but does not occur until 10 days JQ1 treatment. **A** Normalized cell viability of IDH^wt/mut^ glioma cells treated with JQ1 for 10 days (*n* = 3). 2-way ANOVA with multiple comparisons was used for statistical analysis. **B** Time course assessment of normalized cell viability at 3, 7, and 10 days treatment with 250 nM JQ1 (*n* = 3). Statistical analysis was performed using 2-way ANOVA with multiple comparisons. **C** Western blot of cleaved caspase-3 in whole cell lysates extracted from IDH^wt/mut^ glioma cells treated with 250 nM JQ1 for 10 days. **D** Phase-contrast microscopy of IDH^wt/mut^ glioma cells treated with 250 nM JQ1 for 10 days

Next, we performed Muse cell viability assays with the BET inhibitor JQ1 on our IDH^wt^ (0827, 0923) and IDH^mut^ (0905, BT142) cell lines in vitro. To note is that all four cell lines were cultured in defined stem cell media and maintained as glioma stem-like cells. In JQ1 dose–response assays conducted with a 10 day endpoint, our IDH^mut^ cells exhibited a much more pronounced cytotoxic response to JQ1, with IDH^mut^ cells displaying a statistically significant reduction in cell viability relative to IDH^wt^ cells. Reduction in cell viability ranged from 54–72% in IDH^mut^ glioma cells whereas the IDH^wt^ counterpart only had a reduction in cell viability by 10–31% (Fig. 2A; Fig. S1A). Time course assays utilizing 250 nM JQ1 at timepoints of 3, 7, and 10 days showed that a statistically significant cytotoxic response is delayed to 10 days incubation with JQ1 (Fig. 2B; Fig. S1A-C). Further analysis via western blot supported an apoptotic mechanism of cell death in IDH^mut^ cells (Fig. 2C). Phase-contrast microscopy images showed our cells as large and numerous neurospheres in our vehicle treatments, but at 250 nM JQ1 over 10 days we saw preferential reduction in sphere size, sphere number, and an increase in phase-dark single cells in our IDH^mut^ (0905, BT142), but not in our IDH^wt^ (0827, 0923) cultures (Fig. 2D). Overall, these data indicate that IDH^mut^ cells display a profound delayed cytotoxic/apoptotic response that may not be observed in short-term cytotoxicity/apoptosis assays.

### JQ1 early effects are primarily antiproliferative with a modest sensitivity associated with *IDH* status

**Fig. 3:**
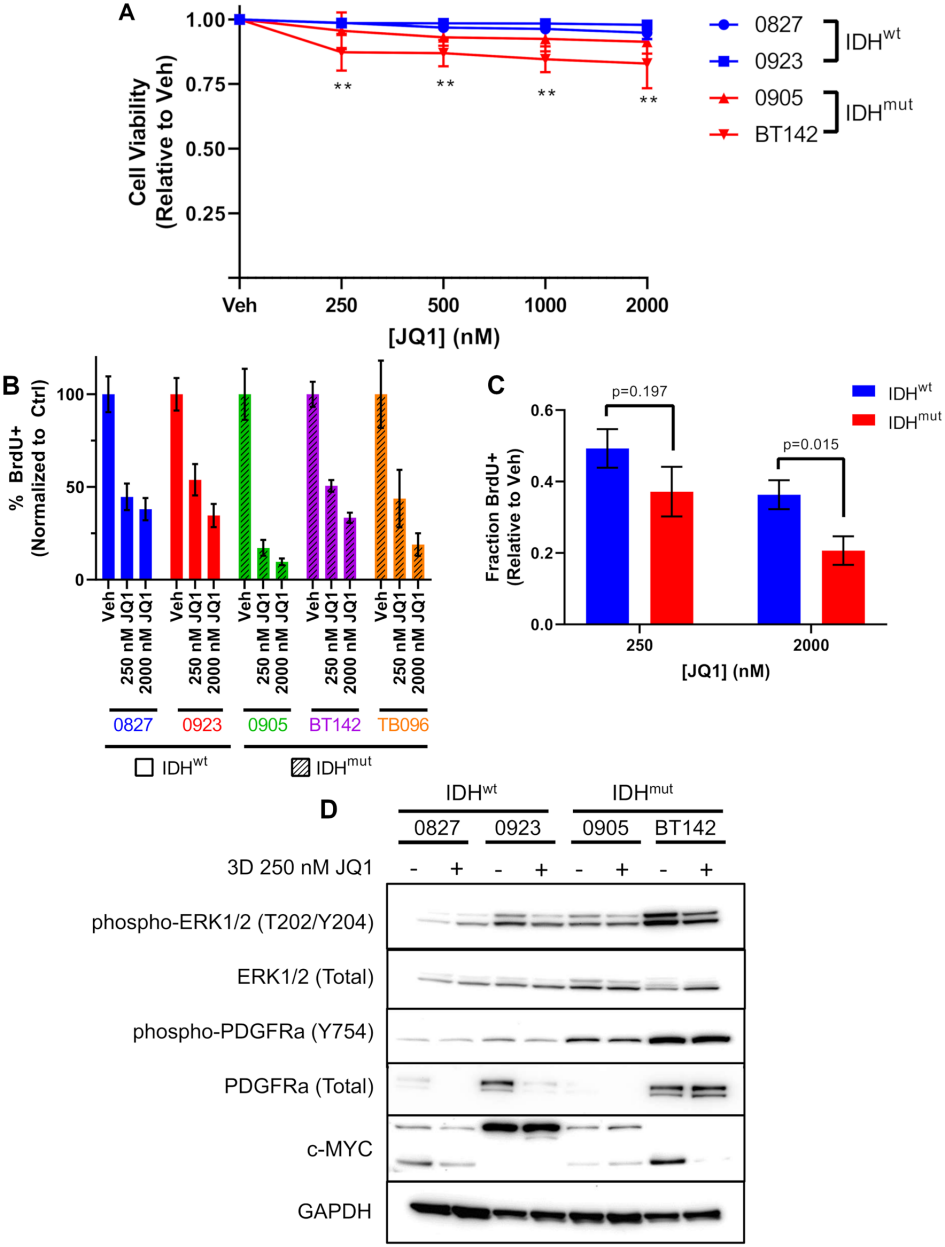
JQ1 early effects are primarily antiproliferative. **A** Cell viability of IDH^wt/mut^ glioma cells treated with JQ1 for 3 days (*n* = 3). Statistical analysis was performed via 2-way ANOVA with multiple comparisons. **B** BrdU incorporation assays of IDH^wt/mut^ glioma treated with 250 nM and 2000 nM JQ1 for 48 h (*n* = 3). **C** Consolidation of data from **B** based on IDH status (*n* = 6). Statistical analysis was performed via Student’s *t*-test. **D** Western blot of ERK1/2, PDGFRa, and c-MYC in whole cell lysates of IDH^wt/mut^ glioma cultures at 3 days JQ1 treatment

Next, we focused on investigating the early effects of JQ1-mediated BET inhibition. We observed that at 3 days incubation with JQ1 there was very little reduction in cell viability irrespective of IDH status (Fig. 3A; Fig. S1C). We then performed BrdU incorporation assays to investigate the antiproliferative effects mediated by JQ1 at this early time point with our IDH^wt/mut^ glioma cells. Notably, we did not see a significant antiproliferative response in our IDH^mut^ cells compared to their wild-type counterpart treated with 250 nM JQ1 for 3 days, with a 50% reduction in BrdU + cells for our IDH^wt^ glioma cultures (0827, 0923) compared to approximately 63% reduction in BrdU + cells for our IDH^mut^ glioma cultures (0905, BT142, TB096) (Fig. 3B, C; Fig. S2A, B). At 2000 nM JQ1 for 3 days, IDH status was associated with a statistically significant, yet modest, decrease in cellular proliferation with a 64% and 80% reduction in BrdU + cells compared to controls for IDH^wt^ and IDH^mut^ glioma cells, respectively (Fig. 3B, C; Fig. S2A, B).

Molecular analysis of parallel samples via western blot showed decreased activation of p-ERK at 3 days incubation with 250 nM JQ1, though these effects were not seen in 0827 (Fig. 3D). Additionally, total PDGFRα levels were downregulated in 0827 and 0923, but no effect on activated p-PDGFRα was seen at this timepoint (Fig. 3D). Effects on c-MYC were not associated with IDH status, with downregulation only seen in 0827 and BT142 GSCs (Fig. 3D).

### Exogenous *R132H IDH1* is sufficient to recapitulate delayed cytotoxicity in response to JQ1

**Fig. 4:**
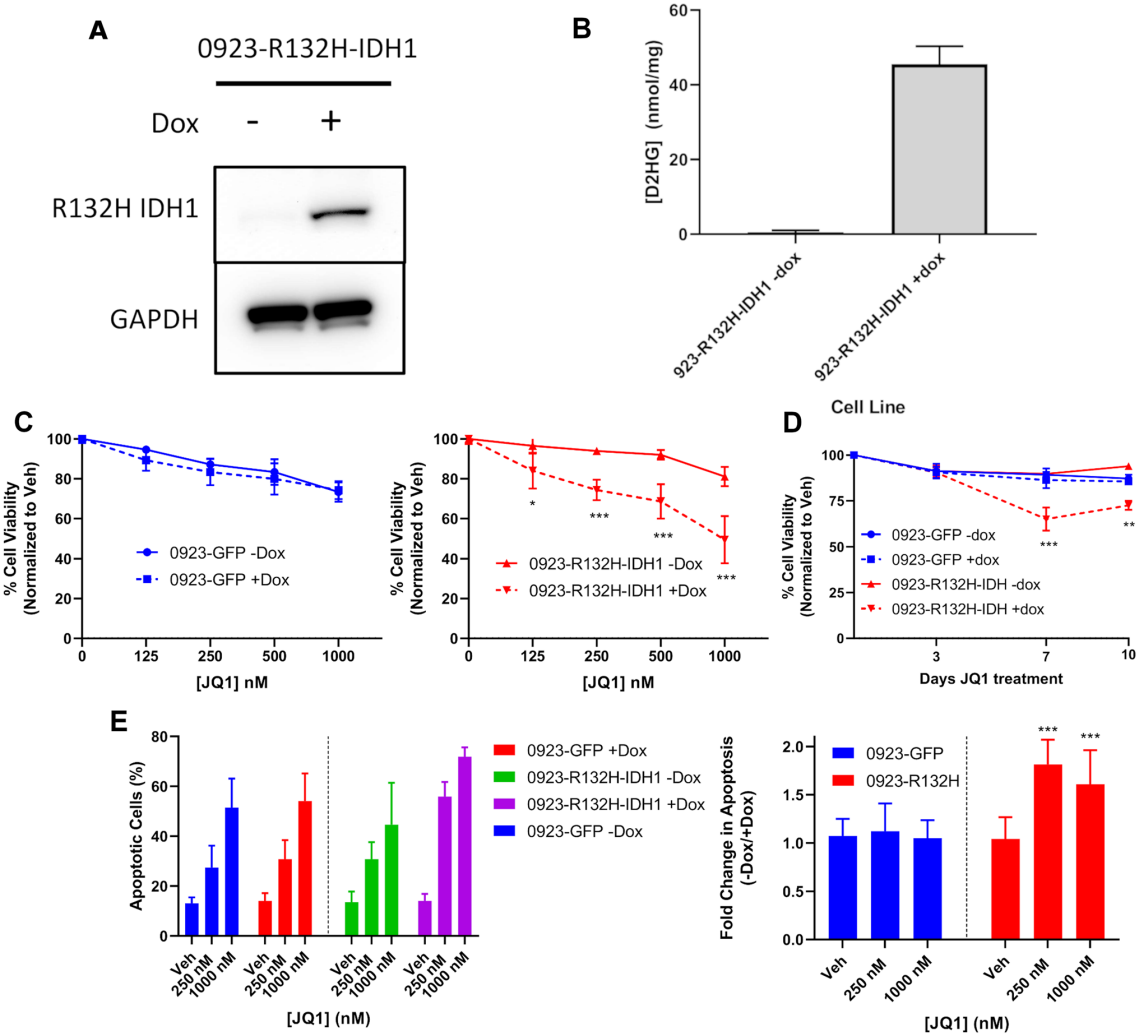
Exogenous R132H IDH1 recapitulates the JQ1-mediated delayed cytotoxicity observed in IDH^mut^ glioma. **A** Validation of R132H IDH1 expression via immunoblot in whole cell lysates of doxycycline-induced 0923-R132H-IDH1. Doxycycline concentration was at or below 0.2 ng/mL. **B** Validation of 2-HG levels by ELISA in doxycycline-induced 0923-R132H-IDH1 (*n* = 3). **C** Loss of cell viability in 0923-GFP and 0923-R132H-IDH1 GBM cells ± doxycycline over 10 days JQ1 treatment (*n* = 3). Statistics were performed via 2-way ANOVA with multiple comparisons. **D** Induction of apoptosis in 0923-GFP and 0923-R132H-IDH1 GBM cells ± doxycycline over 10 days JQ1 treatment (*n* = 3). Statistics were performed via 2-way ANOVA with multiple comparisons. **E** Timecourse assessment of cell viability in 0923-GFP and 0923-R132H GBM cells ± doxycycline at 3, 7, and 10 days treatment with 250 nM JQ1 (*n* = 3). Statistical analysis was performed via 2-way ANOVA with multiple comparisons

To investigate whether *R132H IDH1* is sufficient to sensitize an IDH^wt^ cell line to BET inhibition, we inserted a dox-inducible *R132H IDH1* transgene into our 0923 GBM cell line. Expression of the *R132H IDH1* allele was validated via western blot and elevated 2-HG levels were validated via an enzymatic microplate assay (Fig. 4A, B). We performed a 10-day dose–response cell viability assay with our dox-inducible 0923-R132H-IDH1 cell line ± doxycycline and discovered that induction of *R132H IDH1* expression was sufficient to sensitize 0923 − R132H − IDH1 to the cytotoxic effects of JQ1. In our dose response assay, JQ1 reduced cell viability by 50% for the 0923 − R132H − IDH1 + dox treatment group compared to vehicle controls, whereas cell viability was reduced by only 20% without doxycycline treatment (Fig. 4C; Fig. S3). Similar to our endogenous IDH^mut^ cell lines, the cytotoxic response to JQ1 was delayed past 3 days treatment for 923-R132H-IDH1 + dox, but maximal cytotoxicity appeared to occur earlier at 7 days of treatment (Fig. 4D; Fig. S3). In Annexin V apoptosis assays, *R132H IDH1* transgene expression significantly enhanced the apoptotic response to JQ1 from 31 to 56% at 250 nM and from 45 to 72% at 1000 nM (Fig. 4E; Fig. S4). Doxycycline treatment itself did not elicit any cytotoxic effects at the utilized dose of 0.2 ng/mL. Overall, these data indicate that exogenous *R132H IDH1* is sufficient to potentiate the cytotoxic and apoptotic responses mediated by JQ1 in an IDH^wt^ GBM background.

### Other molecular alterations at late time point

**Fig. 5:**
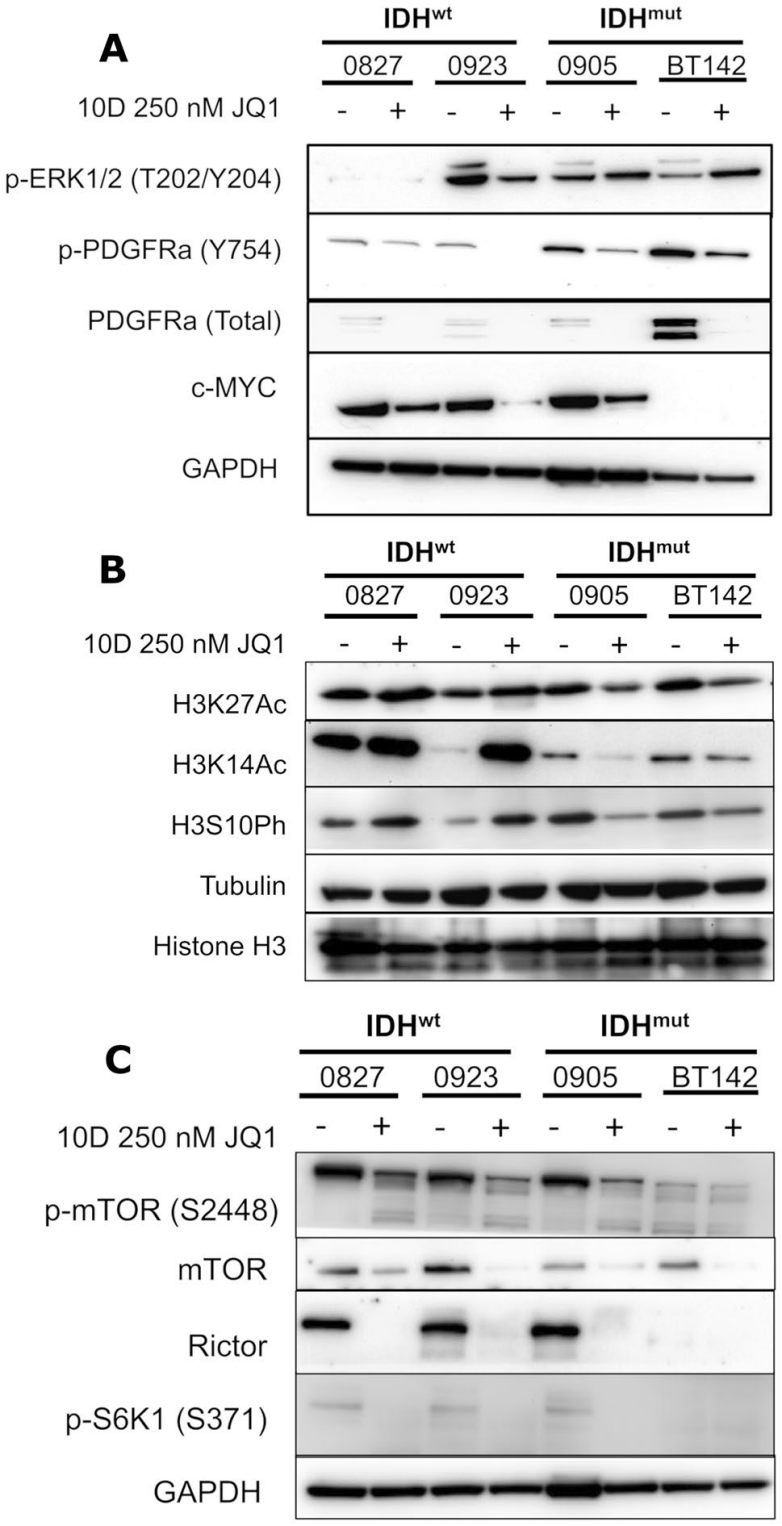
Molecular alterations associated with JQ1 treatment. **A** Western blot of ERK1/2, PDFGRa, and c-MYC in whole cell lysates extracted from IDH^wt/mut^ glioma in response to 250 nM JQ1 for 10 days. **B** Western blot of H3K14Ac, H3K27Ac, and H3S10Ph in whole cell lysates extracted from IDH^wt/mut^ glioma in response to 250 nM JQ1 for 10 days. **C** Western blot of RAPTOR, RICTOR, and mTOR in whole cell lysates extracted from IDH^wt/mut^ glioma in response to 250 nM JQ1 for 10 days. **D** Western blot of RAPTOR, RICTOR, and mTOR in whole cell lysates extracted from IDH^wt/mut^ glioma in response to 250 nM JQ1 for 3 days

We further delved into the molecular responses of IDH^wt/mut^ glioma in response to JQ1 at our late timepoint, 10 days. In contrast to the early 3 day time point, at 10 days JQ1 treatment all cell lines exhibited substantial downregulation of total PDGFRα, and this downregulation was also accompanied by decreased levels of active p-PDGFRα (Fig. 5A). This is especially interesting when considering that only our endogenous IDH^mut^ cells recommend PDGF supplementation in the cell culture media. ERK was also downregulated in all cell lines analyzed, though intriguingly with our IDH^mut^ glioma cells we only see decreased activation of ERK1, but not ERK2 (Fig. 5A). Additionally, we had a more consistent downregulation of c-MYC irrespective of IDH status at 10 days JQ1 treatment, though interestingly expression of c-MYC in BT142 was not observed at this time point (Fig. 5A). Overall, these data support further investigation into the differential role that PDGFRα activation and c-MYC downregulation play in the cytotoxic/antiproliferative response to JQ1.

Additional molecular analysis also showed that certain transcriptionally active epigenetic marks, such as H3K27Ac, H3K14Ac, and H3S10Ph, are downregulated in our IDH^mut^ cells but in their IDH counterparts are conversely upregulated (Fig. 5B). This is interesting as it highlights unique epigenetic responses to BET inhibition in IDH^mut^ glioma, which may owe to the unique epigenetic landscapes in IDH^wt^ vs IDH^mut^ glioma.

Lastly, we observe downregulation of mTORC1/2-related proteins including RAPTOR and RICTOR, with associated decreases in mTOR and S6K phosphorylation in response to 250 nM JQ1 for 10 days (Fig. 5C). To note is that this molecular response is not restricted to IDH^mut^ cell lines. Considering that JQ1-mediated effects on mTORC function have not been reported in the literature, future research in this area could yield a more comprehensive characterization of the molecular effects mediated by BET inhibition.

## Discussion

In this study, we utilized TCGA clinical data combined with cell health assays and showed that IDH^mut^ gliomas are more reliant on the BET transcriptional coactivators for cell survival and proliferation. Analysis of clinical TCGA low grade glioma and glioblastoma data shows BRD4 gene expression correlates with decreased survival only in IDH^mut^ glioma, while BRD3 gene expression correlates with reduced patient survival only in IDH^wt^ glioma. This supports the notion that BET isoform activity promotes malignancy preferentially in IDH^mut^ glioma and suggests that pan-BET inhibitors would be more useful as cancer therapeutics in IDH^mut^ glioma, though our TCGA data also points to the promise of BRD4-selective inhibitors in the treatment of IDH^mut^ glioma. Furthermore, this suggests the clinical benefit of BET inhibitors in the treatment of IDH^wt^ glioma may be limited. Accordingly, a recent Phase II clinical trial with the pan-BET inhibitor OTX015 was withdrawn due to lack of clinical activity (Hottinger et al. 2016).

Previous studies with JQ1 and other BET inhibitors in glioma and other cancers shows that the effects of BET inhibition are primarily antiproliferative while having little effect on cytotoxicity (Pastori et al. 2014; Filippakopoulos et al. 2010; Cheng et al. 2013). Indeed, in cell health studies using JQ1, we observed very little cytotoxicity but moderate antiproliferative effects in IDH^wt^ glioma cells. In contrast, we observed a JQ1-mediated selective cytotoxic response associated with *R132H IDH1* in our endogenous IDH^mut^ glioma cells (0905, BT142) and dox-inducible *R132H IDH1* model that is little observed in the IDH^wt^ counterpart (0827, 0923). Immunoblot and flow cytometric analysis supported an apoptotic mode of cell death in our IDH^mut^ cells. Interestingly, this cytotoxic response is delayed to 10 days incubation with BET inhibitor, with early (3 days) effects of BET inhibition being primarily antiproliferative. This is significant since most in vitro cytotoxicity assays using BET inhibitors utilize endpoints of 3 days or less (Cheng et al. 2013; Filippakopoulos et al. 2010; Pastori et al. 2014), whereas treatment with BET inhibitors in vivo can occur over a period of weeks to months (Pastori et al. 2014; Cheng et al. 2013; Hottinger et al. 2016; Lewin et al. 2018). In addition to the assessments of cytotoxicity, we observed a modest-to-major decrease in cellular proliferation in all cell lines treated with JQ1. We also observed a statistically significant, though modest, preferential decrease in cellular proliferation in our IDH^mut^ glioma cells compared to the IDH^wt^ counterpart. Therefore, our data indicates that IDH^mut^ glioma is preferentially reliant on BET protein function for maintenance of cellular survival and proliferation, and suggests moving onto in vivo models of glioma for further validation of this hypothesis.

Though our molecular data is not conclusive, it presents a potential rationale for the sensitivity of IDH^mut^ glioma to BET inhibition. BRD4 inhibition has been shown to inhibit transcriptional activation of the oncogene c-MYC (Delmore et al. 2011), which has been poised as particularly important for progression from low grade to high grade IDH^mut^ glioma (Bai et al. 2016; Odia et al. 2013). Also interesting are the effects of BET inhibition on PDGFRα activity. There is very little scientific literature regarding BET-mediated regulation of PDGFRα (Wang et al. 2015), but our data suggests that JQ1-mediated PDGFRα downregulation is not uniformly seen at early incubation timepoints (3 days), but becomes more pronounced and accompanied by decreased activation of p-PDGFRα at late timepoints (10 days). This suggests that though total PDGFRα downregulation may occur early in the time course of JQ1 treatment, the functional effects of PDGFRα downregulation are not observed until later during JQ1 treatment. Additionally, IDH^mut^ has been shown to activate PDGFRα signaling via epigenetic silencing of CTCF activity (Flavahan et al. 2016), and in our cell cultures PDGF-AB is supplemented in culture medium only in IDH^mut^ cells (0905, BT142). However, we were not able to ascertain the specific role decreased PDGFRα activation played in the cytotoxic and antiproliferative responses of our glioma cells to JQ1.

Additional molecular analysis revealed a selective downregulation of various active chromatin marks, notably H3K14/27Ac (Karmodiya et al. 2012; Creyghton et al. 2010) and H3S10Ph (Zippo et al. 2009; Karam et al. 2010), only in our IDH^mut^ glioma cells. This is particularly interesting as it suggests selective epigenetic, and therefore transcriptional, effects in our IDH^mut^ glioma cells. Through our limited molecular analysis via immunoblotting, we were unable to find any selective upregulation or downregulation of pathways that would corroborate selective transcriptional alterations in our IDH^mut^ glioma cells. This supports further analysis with more comprehensive, unbiased molecular techniques such as ATAC-Seq or ChIP-Seq, coupled with RNA-Seq, to identify unique changes in transcriptional programs in IDH^wt^ vs IDH^mut^ glioma in response to BET inhibition.

In this study, we provide clinical and in vitro evidence that IDH^mut^ glioma exhibits a preferential reliance on BET protein activity to maintain cellular survival, proliferation, and tumor malignancy. Considering BET inhibitors have had limited success in the clinic, this study suggests repurposing these BET inhibitors for the treatment of IDH^mut^ glioma may yield a promising therapeutic strategy.

## Supplementary Information

Below is the link to the electronic supplementary material.

Supplementary file1 (PDF 1852 KB)

Supplementary file2 (PDF 1314 KB)

Supplementary file3 (PDF 778 KB)

## Data Availability

The datasets generated during and/or analysed during the current study are available from the corresponding author on request.

## References

[CR1] Bai H, Harmancı AS, Erson-Omay EZ, Li J, Coşkun S, Simon M, Krischek B, Özduman K, Omay SB, Sorensen EA, Turcan Ş, Bakırcığlu M, Carrión-Grant G, Murray PB, Clark VE, Ercan-Sencicek AG, Knight J, Sencar L, Altınok S, Kaulen LD, Gülez B, Timmer M, Schramm J, Mishra-Gorur K, Henegariu O, Moliterno J, Louvi A, Chan TA, Tannheimer SL, Pamir MN, Vortmeyer AO, Bilguvar K, Yasuno K, Günel M (2016) Integrated genomic characterization of IDH1-mutant glioma malignant progression. Nat Genet 48:59–66. 10.1038/ng.3457 26618343 10.1038/ng.3457 PMC4829945

[CR2] Belkina AC, Nikolajczyk BS, Denis GV (2013) BET protein function is required for inflammation: Brd2 genetic disruption and BET inhibitor JQ1 impair mouse macrophage inflammatory responses. J Immunol 190:3670–3678. 10.4049/jimmunol.1202838 23420887 10.4049/jimmunol.1202838 PMC3608815

[CR3] Bolden JE, Tasdemir N, Dow LE, van Es JH, Wilkinson JE, Zhao Z, Clevers H, Lowe SW (2014) Inducible in vivo silencing of Brd4 identifies potential toxicities of sustained BET protein inhibition. Cell Rep 8:1919–1929. 10.1016/j.celrep.2014.08.025 25242322 10.1016/j.celrep.2014.08.025 PMC4234106

[CR4] Cheng Z, Gong Y, Ma Y, Lu K, Lu X, Pierce LA, Thompson RC, Muller S, Knapp S, Wang J (2013) Inhibition of BET bromodomain targets genetically diverse glioblastoma. Clin Cancer Res 19:1748. 10.1158/1078-0432.CCR-12-3066 23403638 10.1158/1078-0432.CCR-12-3066 PMC4172367

[CR5] Creyghton MP, Cheng AW, Welstead GG, Kooistra T, Carey BW, Steine EJ, Hanna J, Lodato MA, Frampton GM, Sharp PA, Boyer LA, Young RA, Jaenisch R (2010) Histone H3K27ac separates active from poised enhancers and predicts developmental state. Proc Natl Acad Sci 107:21931–21936. 10.1073/pnas.1016071107 21106759 10.1073/pnas.1016071107 PMC3003124

[CR6] Delmore JE, Issa GC, Lemieux ME, Rahl PB, Shi J, Jacobs HM, Kastritis E, Gilpatrick T, Paranal RM, Qi J (2011) BET bromodomain inhibition as a therapeutic strategy to target c-Myc. Cell 146:904–917. 10.1016/j.cell.2011.08.017 21889194 10.1016/j.cell.2011.08.017 PMC3187920

[CR7] Ene CI, Edwards L, Riddick G, Baysan M, Woolard K, Kotliarova S, Lai C, Belova G, Cam M, Walling J (2012) Histone demethylase Jumonji D3 (JMJD3) as a tumor suppressor by regulating p53 protein nuclear stabilization. PLoS ONE. 10.1371/journal.pone.0051407 23236496 10.1371/journal.pone.0051407 PMC3517524

[CR8] Filippakopoulos P, Qi J, Picaud S, Shen Y, Smith WB, Fedorov O, Morse EM, Keates T, Hickman TT, Felletar I, Philpott M, Munro S, McKeown MR, Wang Y, Christie AL, West N, Cameron MJ, Schwartz B, Heightman TD, La Thangue N, French CA, Wiest O, Kung AL, Knapp S, Bradner JE (2010) Selective inhibition of BET bromodomains. Nature 468:1067–1073. 10.1038/nature09504 20871596 10.1038/nature09504 PMC3010259

[CR9] Flavahan WA, Drier Y, Liau BB, Gillespie SM, Venteicher AS, Stemmer-Rachamimov AO, Suvà ML, Bernstein BE (2016) Insulator dysfunction and oncogene activation in IDH mutant gliomas. Nature 529:110–114. 10.1038/nature16490 26700815 10.1038/nature16490 PMC4831574

[CR10] Fujiwara H, Tateishi K, Kato H, Nakatsuka T, Yamamoto K, Tanaka Y, Ijichi H, Takahara N, Mizuno S, Kogure H (2018) Isocitrate dehydrogenase 1 mutation sensitizes intrahepatic cholangiocarcinoma to the BET inhibitor JQ1. Cancer Sci 109:3602–3610. 10.1111/cas.13784 30156013 10.1111/cas.13784 PMC6215870

[CR11] Goldman MJ, Craft B, Hastie M, Repečka K, McDade F, Kamath A, Banerjee A, Luo Y, Rogers D, Brooks AN (2020) Visualizing and interpreting cancer genomics data via the Xena platform. Nat Biotechnol. 10.1038/s41587-020-0546-8 32444850 10.1038/s41587-020-0546-8 PMC7386072

[CR12] Horne GA, Stewart HJS, Dickson J, Knapp S, Ramsahoye B, Chevassut T (2015) Nanog requires BRD4 to maintain murine embryonic stem cell pluripotency and is suppressed by bromodomain inhibitor JQ1 together with Lefty1. Stem Cells Dev 24:879–891. 10.1089/scd.2014.0302 25393219 10.1089/scd.2014.0302 PMC4367495

[CR13] Hottinger AF, Sanson M, Moyal E, Delord J-P, De Micheli R, Rezai K, Leung ACF, Perez S, Bekradda M, Lachaux N, Lokiec FM, Chinot OL (2016) Dose optimization of MK-8628 (OTX015), a small molecule inhibitor of bromodomain and extra-terminal (BET) proteins, in patients (pts) with recurrent glioblastoma (GB). J Clin Oncol 34:e14123–e14123. 10.1200/JCO.2016.34.15_suppl.e14123

[CR14] Jain AK, Barton MC (2017) Bromodomain histone readers and cancer. J Mol Biol 429:2003–2010. 10.1016/j.jmb.2016.11.020 27890782 10.1016/j.jmb.2016.11.020

[CR15] Karam CS, Kellner WA, Takenaka N, Clemmons AW, Corces VG (2010) 14–3-3 mediates histone cross-talk during transcription elongation in Drosophila. PLoS Genet. 10.1371/journal.pgen.1000975 20532201 10.1371/journal.pgen.1000975 PMC2880557

[CR16] Karmodiya K, Krebs AR, Oulad-Abdelghani M, Kimura H, Tora L (2012) H3K9 and H3K14 acetylation co-occur at many gene regulatory elements, while H3K14ac marks a subset of inactive inducible promoters in mouse embryonic stem cells. BMC Genomics 13:424. 10.1186/1471-2164-13-424 22920947 10.1186/1471-2164-13-424 PMC3473242

[CR17] Korshunov A, Casalini B, Chavez L, Hielscher T, Sill M, Ryzhova M, Sharma T, Schrimpf D, Stichel D, Capper D (2019) Integrated molecular characterization of IDH-mutant glioblastomas. Neuropathol Appl Neurobiol 45:108–118. 10.1111/nan.12523 30326163 10.1111/nan.12523

[CR18] Lewin J, Soria J-C, Stathis A, Delord J-P, Peters S, Awada A, Aftimos PG, Bekradda M, Rezai K, Zeng Z (2018) Phase Ib trial with birabresib, a small-molecule inhibitor of bromodomain and extraterminal proteins, in patients with selected advanced solid tumors. J Clin Oncol 36:3007–3014. 10.1200/JCO.2018.78.2292 29733771 10.1200/JCO.2018.78.2292

[CR19] Luchman HA, Stechishin OD, Dang NH, Blough MD, Chesnelong C, Kelly JJ, Nguyen SA, Chan JA, Weljie AM, Cairncross JG (2011) An in vivo patient-derived model of endogenous IDH1-mutant glioma. Neuro Oncol 14:184–191. 10.1093/neuonc/nor207 22166263 10.1093/neuonc/nor207 PMC3266388

[CR20] Mazor T, Chesnelong C, Pankov A, Jalbert LE, Hong C, Hayes J, Smirnov IV, Marshall R, Souza CF, Shen Y, Viswanath P, Noushmehr H, Ronen SM, Jones SJM, Marra MA, Cairncross JG, Perry A, Nelson SJ, Chang SM, Bollen AW, Molinaro AM, Bengtsson H, Olshen AB, Weiss S, Phillips JJ, Luchman HA, Costello JF (2017) Clonal expansion and epigenetic reprogramming following deletion or amplification of mutant IDH1. Proc Natl Acad Sci USA 114:10743–10748. 10.1073/pnas.1708914114 28916733 10.1073/pnas.1708914114 PMC5635900

[CR21] McCleland ML, Mesh K, Lorenzana E, Chopra VS, Segal E, Watanabe C, Haley B, Mayba O, Yaylaoglu M, Gnad F, Firestein R (2016) CCAT1 is an enhancer-templated RNA that predicts BET sensitivity in colorectal cancer. J Clin Invest 126:639–652. 10.1172/jci83265 26752646 10.1172/JCI83265 PMC4731162

[CR22] Miller TE, Liau BB, Wallace LC, Morton AR, Xie Q, Dixit D, Factor DC, Kim LJY, Morrow JJ, Wu Q, Mack SC, Hubert CG, Gillespie SM, Flavahan WA, Hoffmann T, Thummalapalli R, Hemann MT, Paddison PJ, Horbinski CM, Zuber J, Scacheri PC, Bernstein BE, Tesar PJ, Rich JN (2017) Transcription elongation factors represent in vivo cancer dependencies in glioblastoma. Nature 547:355–359. 10.1038/nature23000 28678782 10.1038/nature23000 PMC5896562

[CR23] Moure CJ, Diplas BH, Chen LH, Yang R, Pirozzi CJ, Wang Z, Spasojevic I, Waitkus MS, He Y, Yan H (2019) CRISPR editing of mutant IDH1 R132H induces a CpG methylation-low state in patient-derived glioma models of G-CIMP. Mol Cancer Res 17:2042–2050. 10.1158/1541-7786.MCR-19-0309 31292202 10.1158/1541-7786.MCR-19-0309 PMC6774824

[CR24] Odia Y, Orr BA, Bell WR, Eberhart CG, Rodriguez FJ (2013) cMYC expression in infiltrating gliomas: associations with IDH1 mutations, clinicopathologic features and outcome. J Neurooncol 115:249–259. 10.1007/s11060-013-1221-4 23934175 10.1007/s11060-013-1221-4 PMC3881260

[CR25] Pastori C, Daniel M, Penas C, Volmar C-H, Johnstone AL, Brothers SP, Graham RM, Allen B, Sarkaria JN, Komotar RJ (2014) BET bromodomain proteins are required for glioblastoma cell proliferation. Epigenetics 9:611–620. 10.4161/epi.27906 24496381 10.4161/epi.27906 PMC4121371

[CR26] Sanson M, Marie Y, Paris S, Idbaih A, Laffaire J, Ducray F, El Hallani S, Boisselier B, Mokhtari K, Hoang-Xuan K (2009) Isocitrate dehydrogenase 1 codon 132 mutation is an important prognostic biomarker in gliomas. J Clin Oncol 27:4150–4154. 10.1200/JCO.2009.21.9832 19636000 10.1200/JCO.2009.21.9832

[CR27] Sears TK, Horbinski CM, Woolard KD (2021) IDH1 mutant glioma is preferentially sensitive to the HDAC inhibitor panobinostat. J Neuro-Oncol. 10.1007/s11060-021-03829-0 10.1007/s11060-021-03829-0 PMC8437887 34424450

[CR28] Son MJ, Woolard K, Nam D-H, Lee J, Fine HA (2009) SSEA-1 is an enrichment marker for tumor-initiating cells in human glioblastoma. Cell Stem Cell 4:440–452. 10.1016/j.stem.2009.03.003 19427293 10.1016/j.stem.2009.03.003 PMC7227614

[CR29] Taniguchi Y (2016) The bromodomain and extra-terminal domain (BET) family: functional anatomy of BET paralogous proteins. Int J Mol Sci 17:1849. 10.3390/ijms17111849 27827996 10.3390/ijms17111849 PMC5133849

[CR30] Toledo CM, Ding Y, Hoellerbauer P, Davis RJ, Basom R, Girard EJ, Lee E, Corrin P, Hart T, Bolouri H, Davison J, Zhang Q, Hardcastle J, Aronow BJ, Plaisier CL, Baliga NS, Moffat J, Lin Q, Li X-N, Nam D-H, Lee J, Pollard SM, Zhu J, Delrow JJ, Clurman BE, Olson JM, Paddison PJ (2015) Genome-wide CRISPR-Cas9 screens reveal loss of redundancy between PKMYT1 and WEE1 in glioblastoma stem-like cells. Cell Rep 13:2425–2439. 10.1016/j.celrep.2015.11.021 26673326 10.1016/j.celrep.2015.11.021 PMC4691575

[CR31] Wang B, Zhang M, Takayama T, Shi X, Roenneburg DA, Kent KC, Guo L-W (2015) BET bromodomain blockade mitigates intimal hyperplasia in rat carotid arteries. EBioMedicine 2:1650–1661. 10.1016/j.ebiom.2015.09.045 26870791 10.1016/j.ebiom.2015.09.045 PMC4740308

[CR32] Wu T, Pinto HB, Kamikawa YF, Donohoe ME (2015) The BET family member BRD4 interacts with OCT4 and regulates pluripotency gene expression. Stem Cell Reports 4:390–403. 10.1016/j.stemcr.2015.01.012 25684227 10.1016/j.stemcr.2015.01.012 PMC4375790

[CR33] Xu L, Chen Y, Mayakonda A, Koh L, Chong YK, Buckley DL, Sandanaraj E, Lim SW, Lin RY-T, Ke X-Y (2018) Targetable BET proteins-and E2F1-dependent transcriptional program maintains the malignancy of glioblastoma. Proc Natl Acad Sci 115:E5086–E5095. 10.1073/pnas.1712363115 29764999 10.1073/pnas.1712363115 PMC5984485

[CR34] Zippo A, Serafini R, Rocchigiani M, Pennacchini S, Krepelova A, Oliviero S (2009) Histone crosstalk between H3S10ph and H4K16ac generates a histone code that mediates transcription elongation. Cell 138:1122–1136. 10.1016/j.cell.2009.07.031 19766566 10.1016/j.cell.2009.07.031

