# Supplementary figures and images for: R132H IDH1 sensitizes glioma to the antiproliferative and cytotoxic effects of BET inhibition
Source: J Cancer Res Clin Oncol. 2022 Apr 25;148(9):2275–85. doi: 10.1007/s00432-022-04018-w (PMC9349147; doi:10.1007/s00432-022-04018-w)

# [JQ1] (nM)

**A**

0827

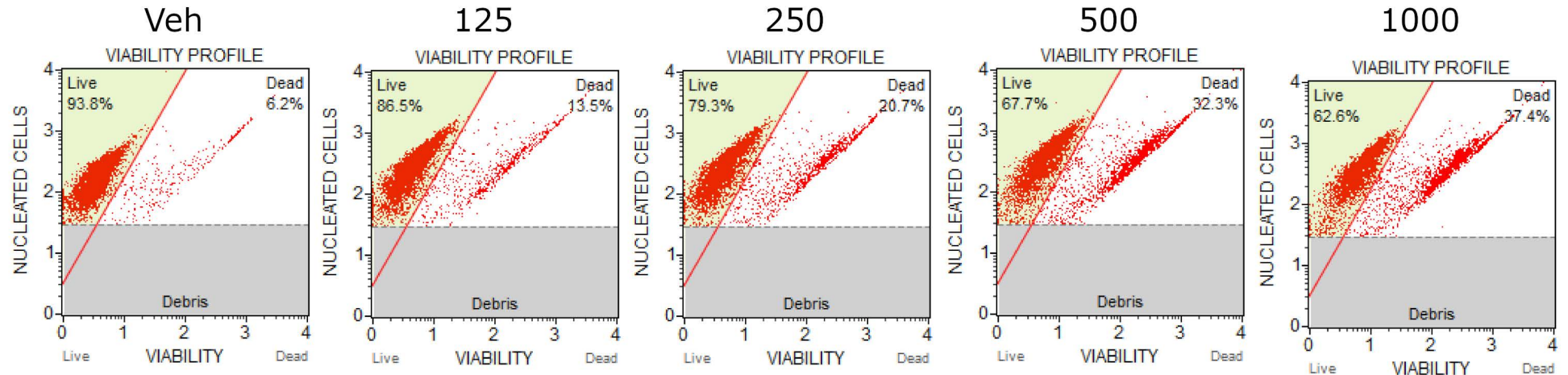

0923

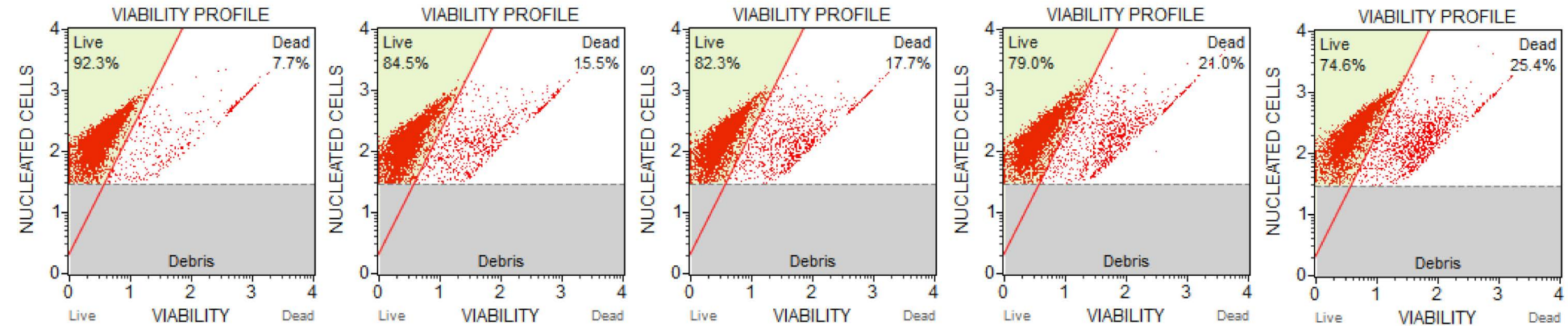

0905

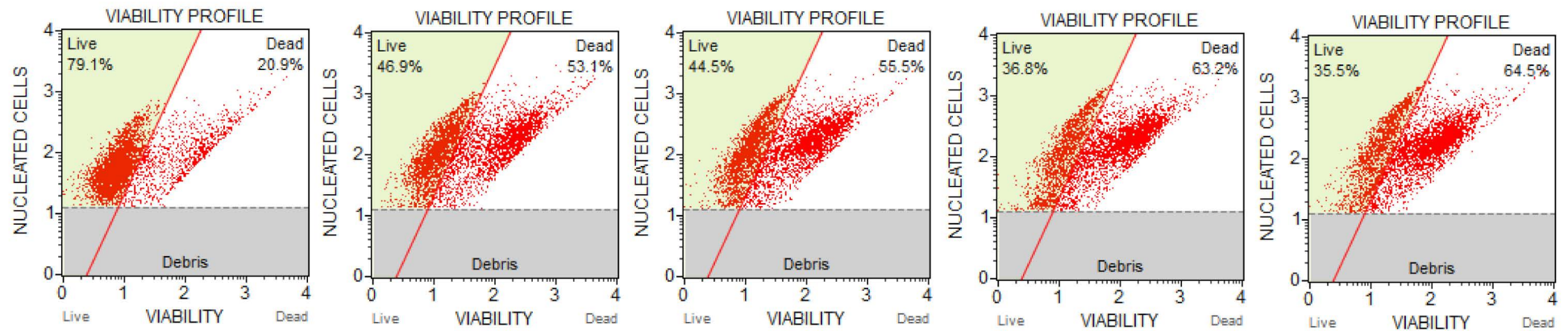

BT142

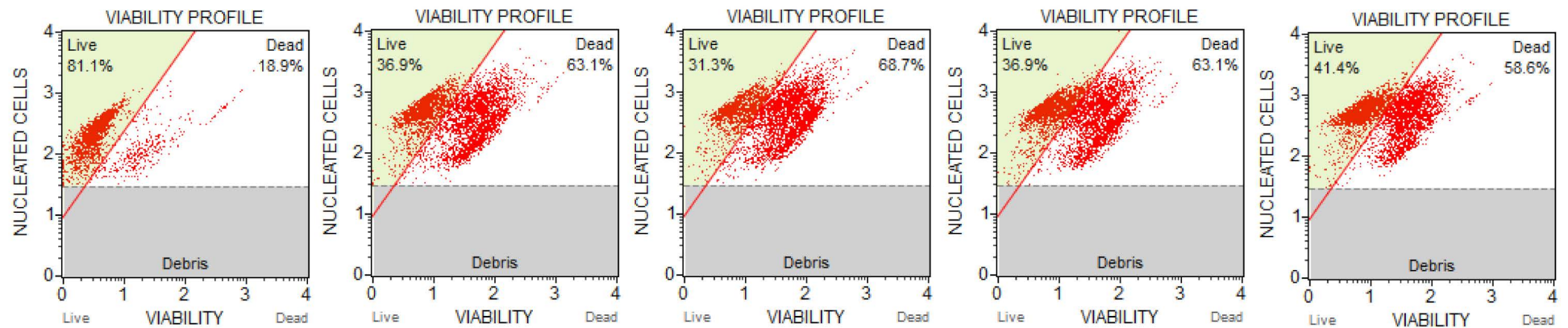

[JQ1] (nM)

**B**

Veh

250

0827

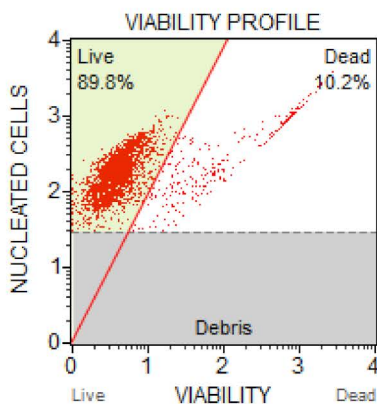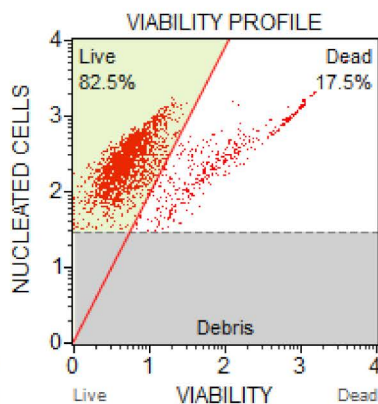

0923

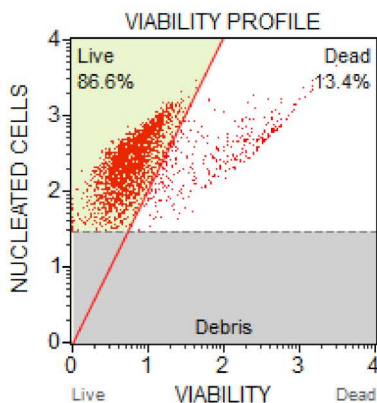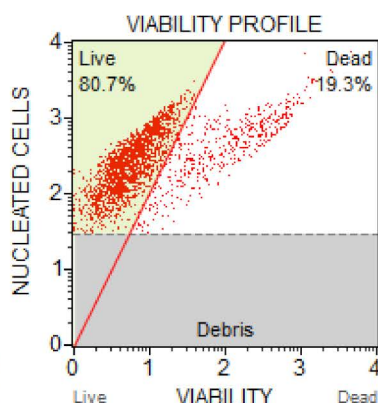

0905

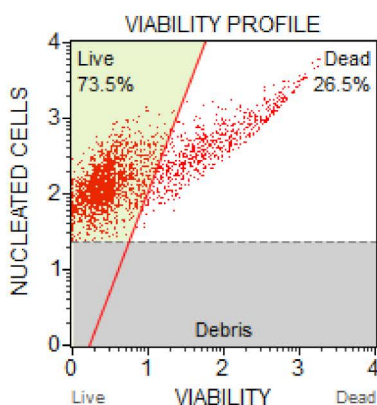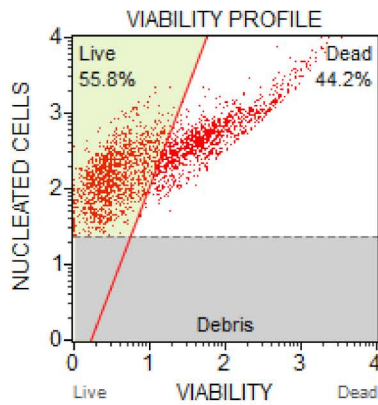

BT142

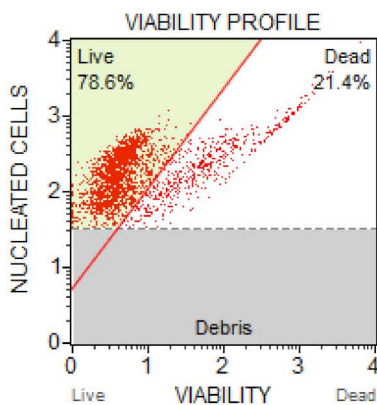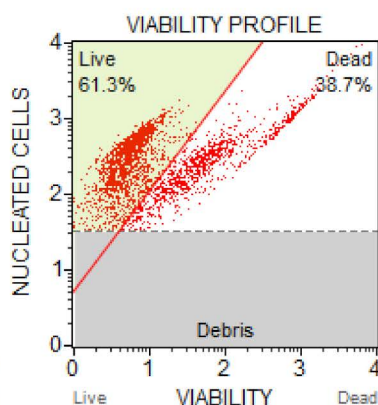

# [JQ1] (nM)

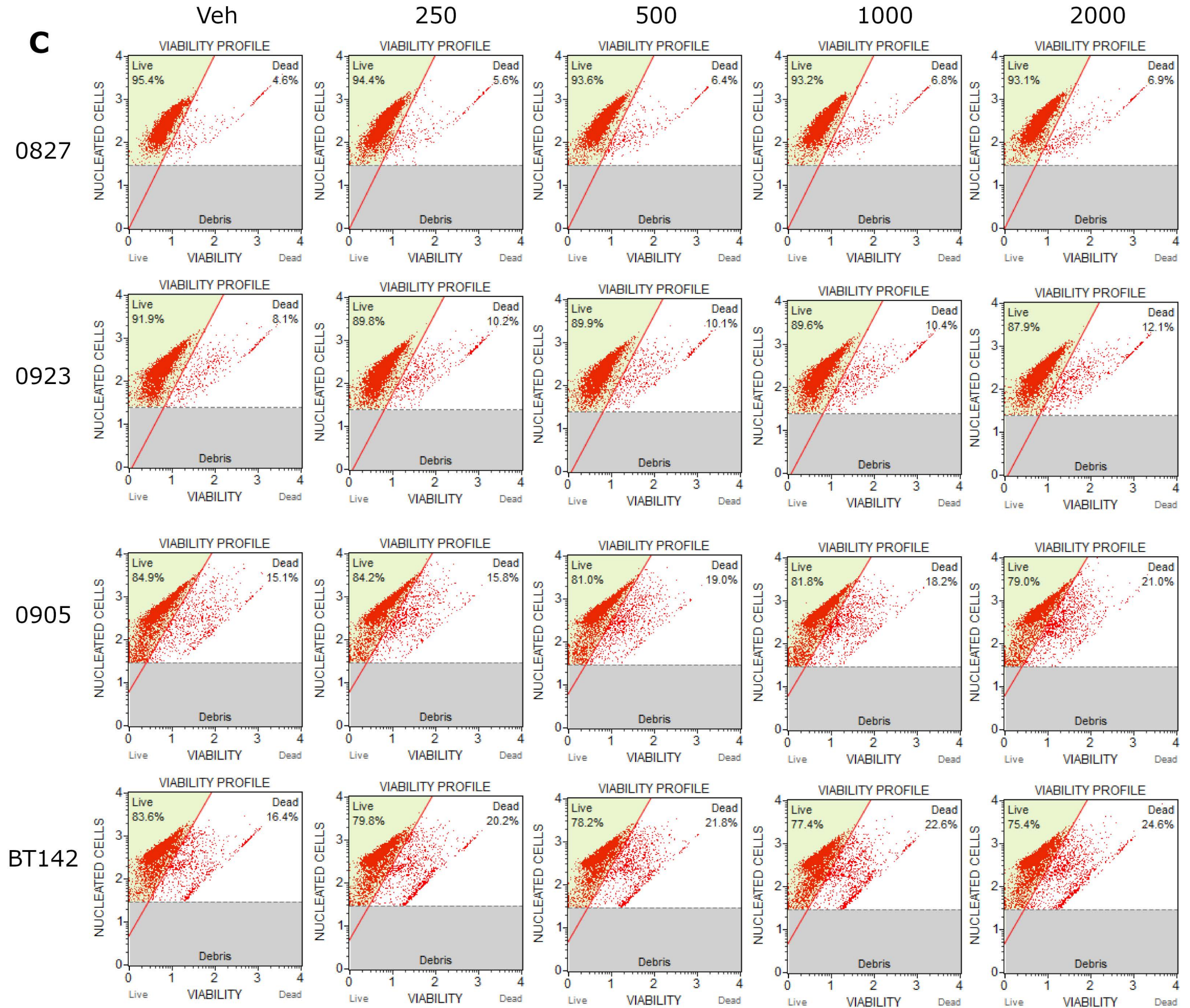

Supplement: Supplementary file 1 — Supplementary file1 (PDF 1852 KB) [file 432_2022_4018_MOESM1_ESM.pdf]

# [JQ1] (nM)

**A**

BC

Veh

250

2000

0827

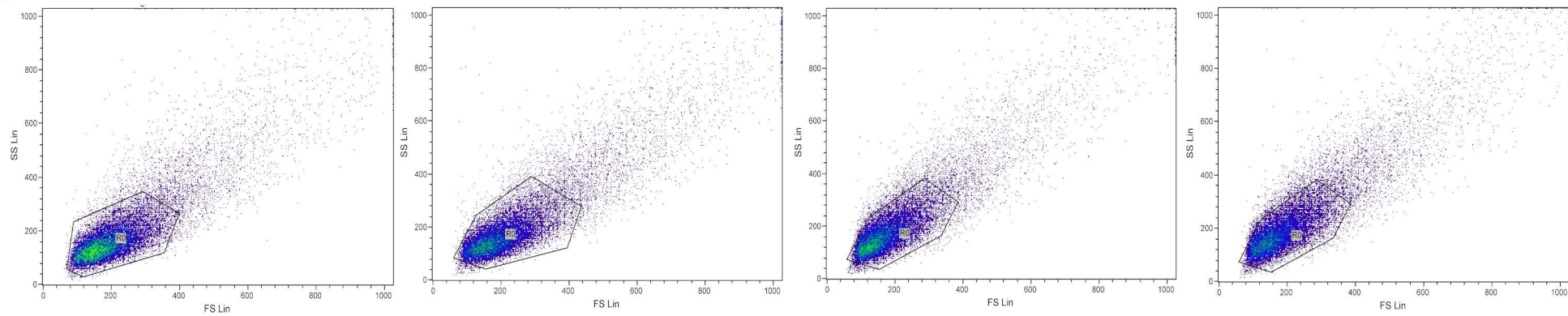

0923

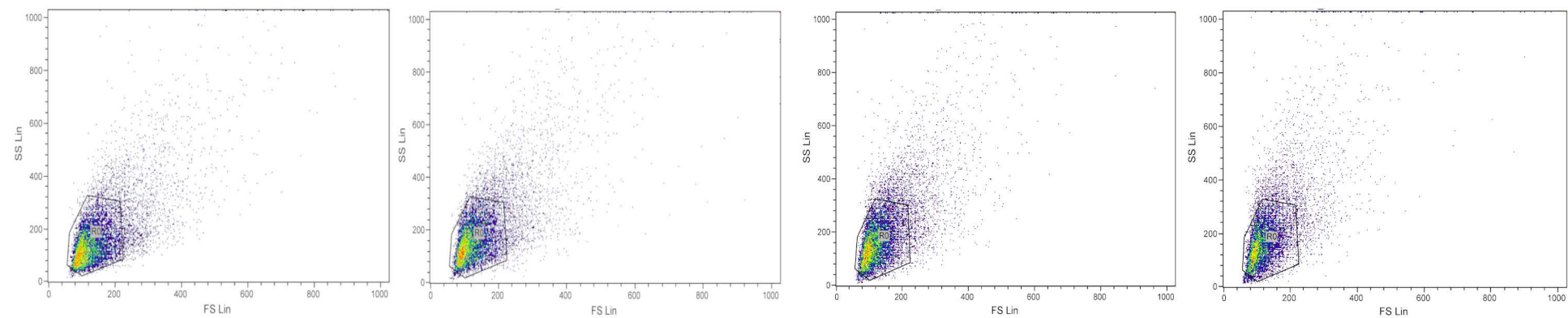

0905

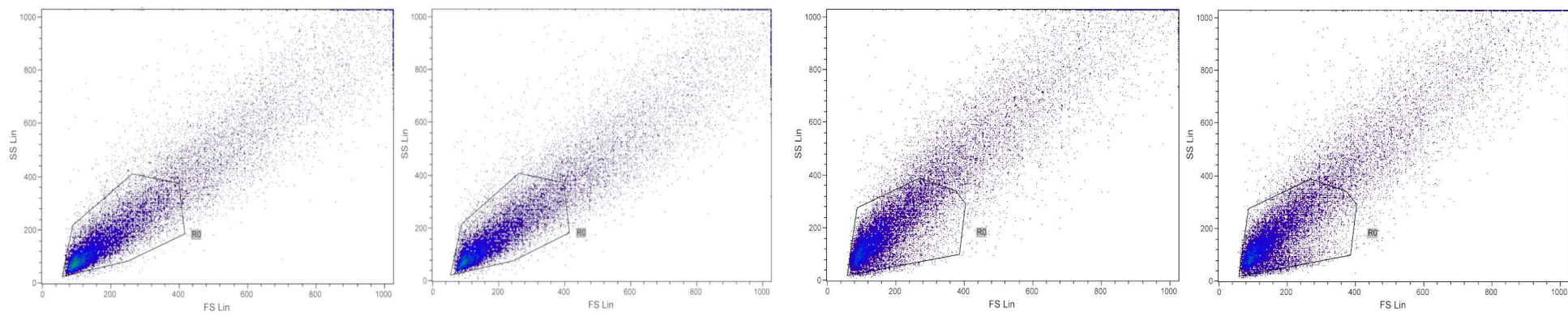

BT142

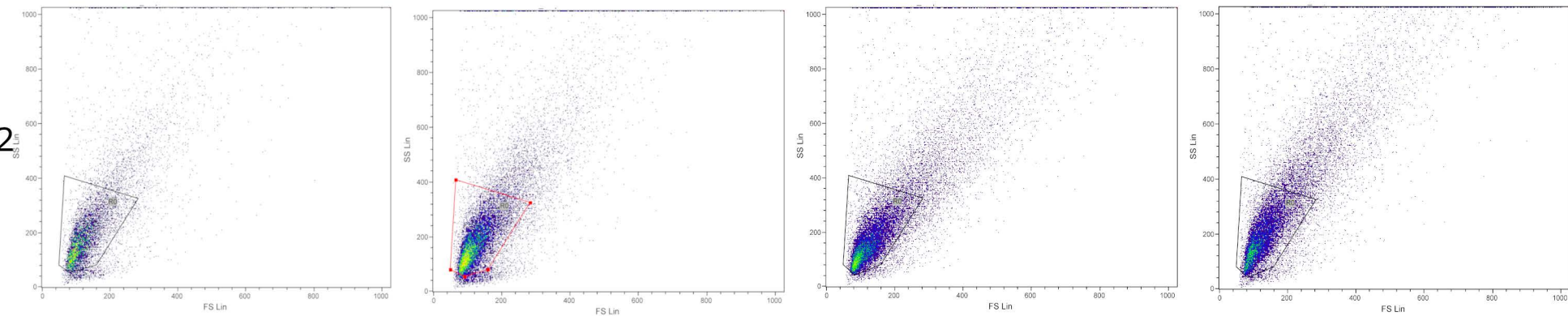

TB096

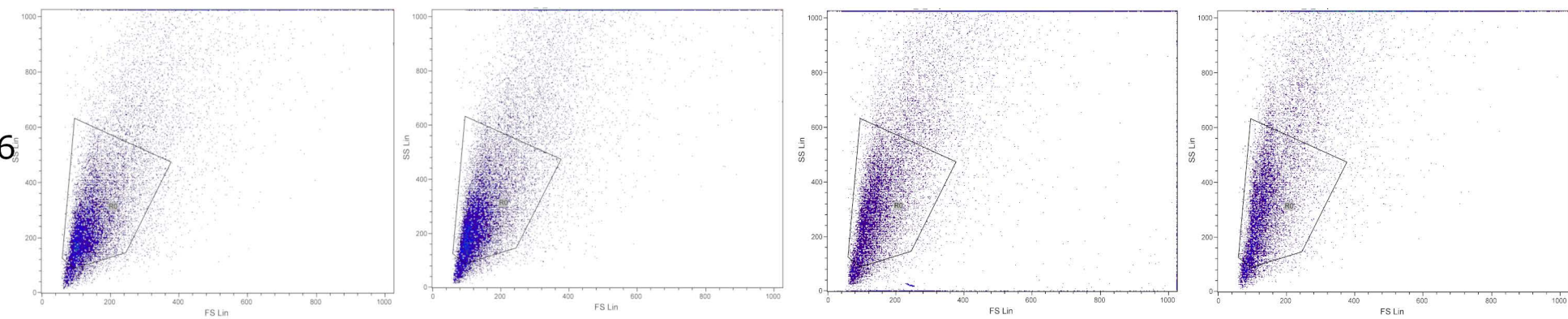

[JQ1] (nM)

B

BC

Veh

250

2000

0827

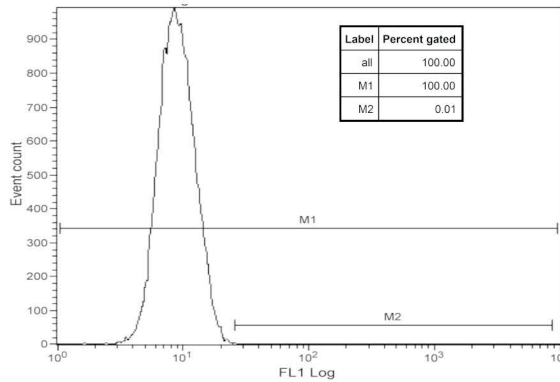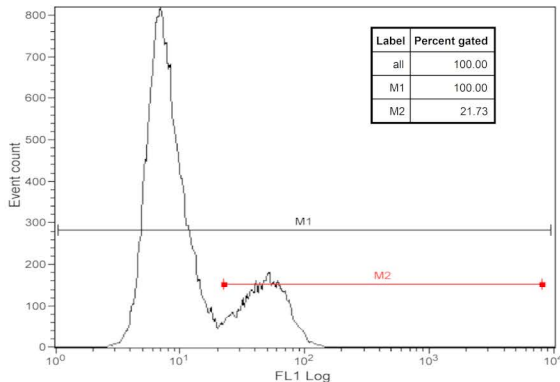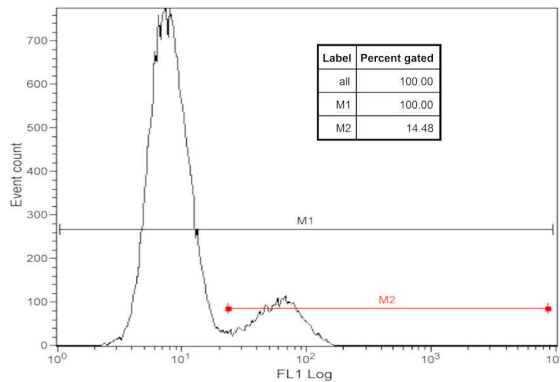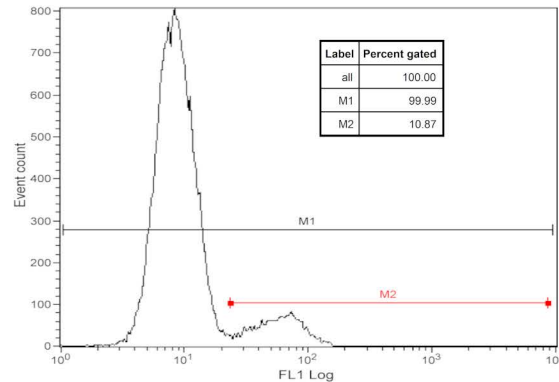

0923

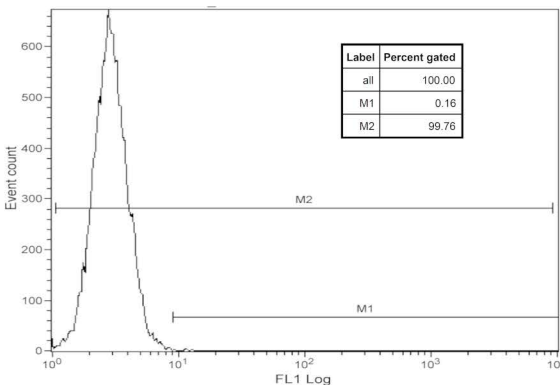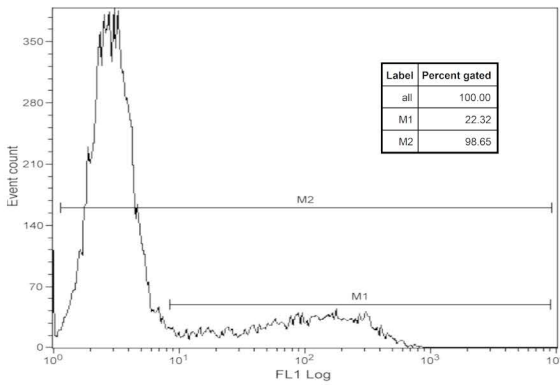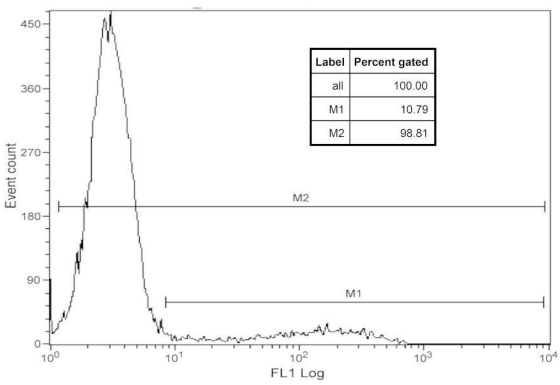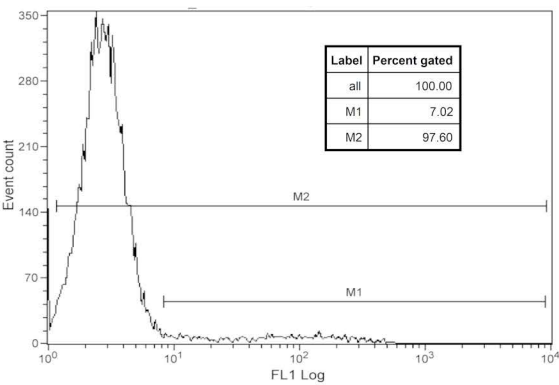

0905

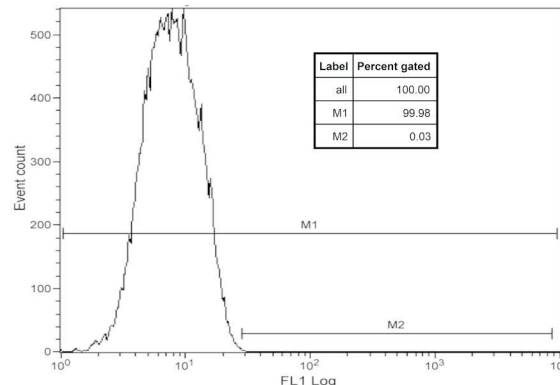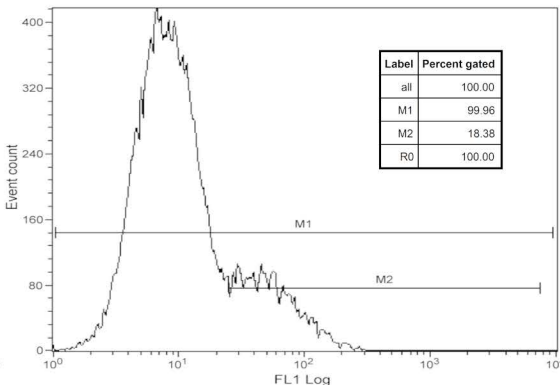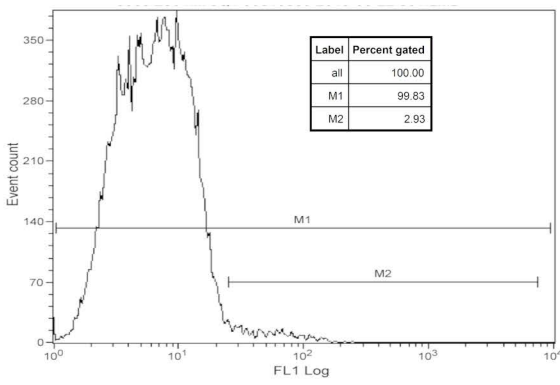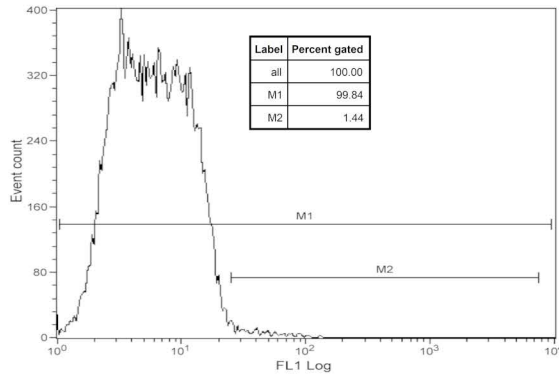

BT142

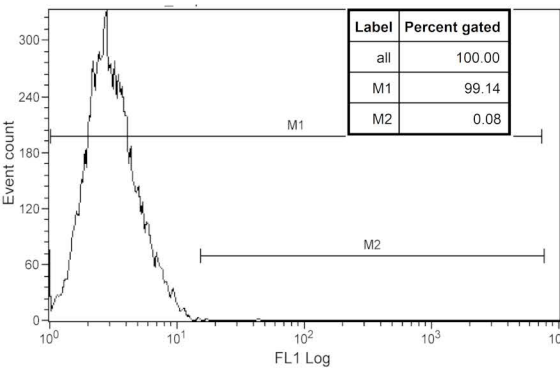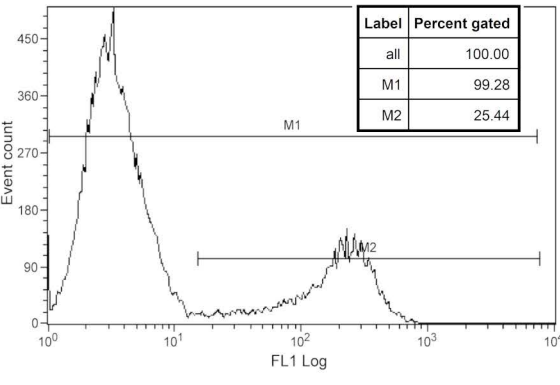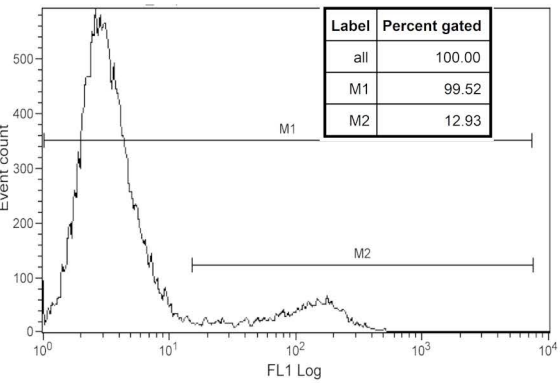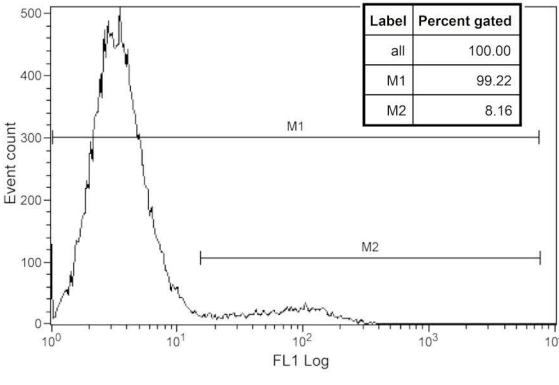

TB096

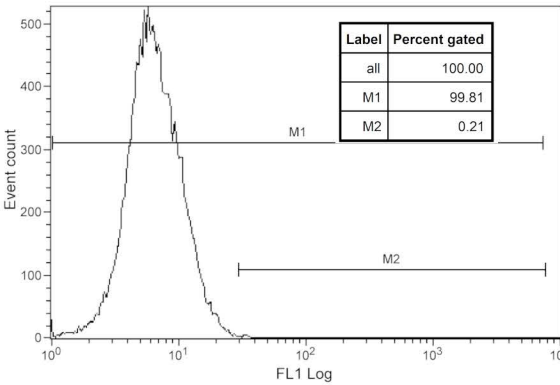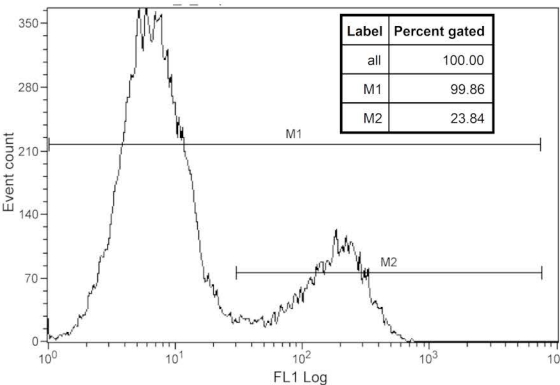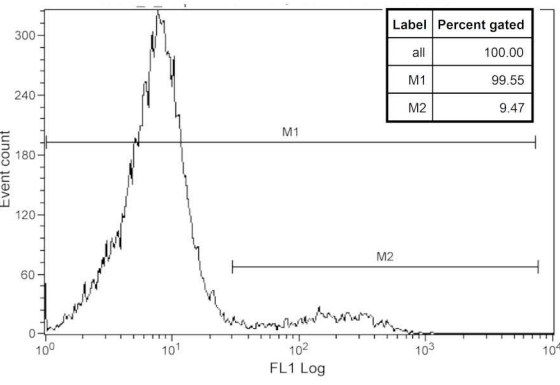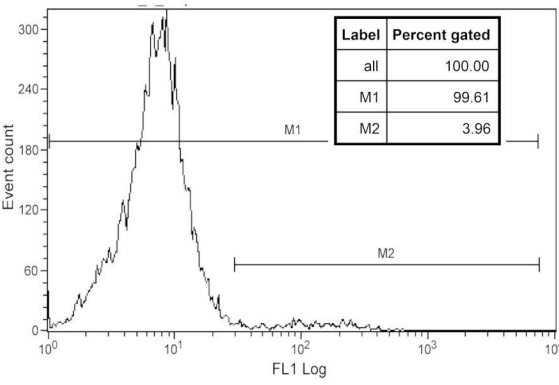

Supplement: Supplementary file 2 — Supplementary file2 (PDF 1314 KB) [file 432_2022_4018_MOESM2_ESM.pdf]

# [JQ1] (nM)

Veh

125

250

500

1000

0923-GFP  
-dox

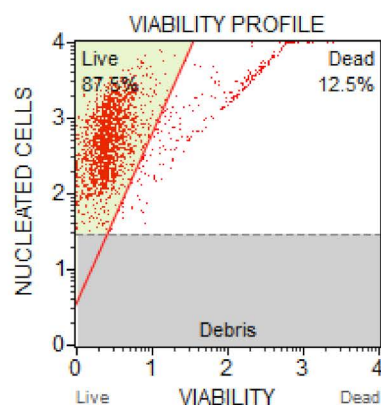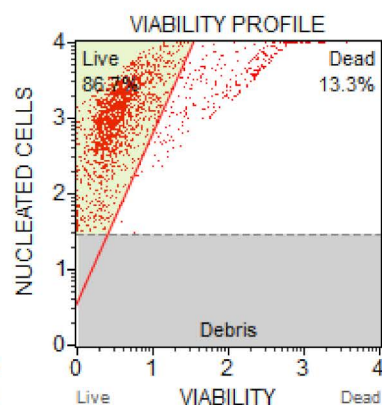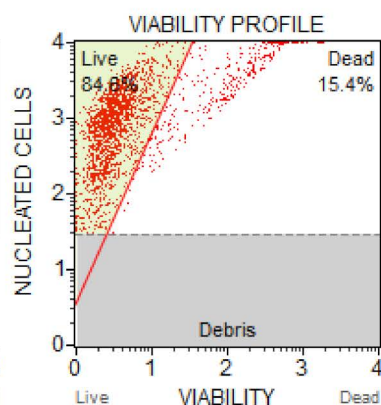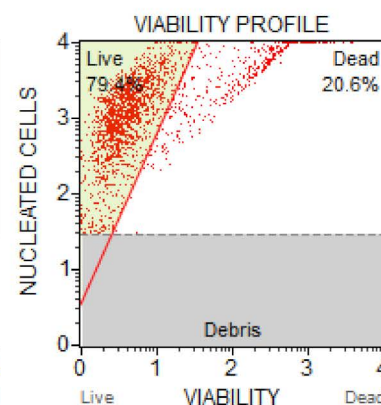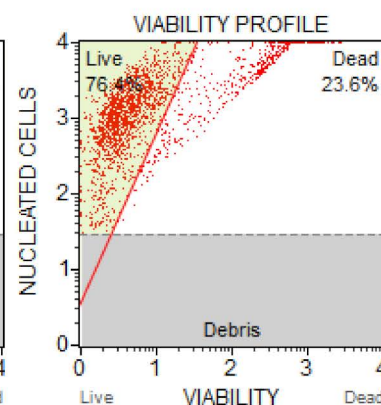

0923-GFP  
+dox

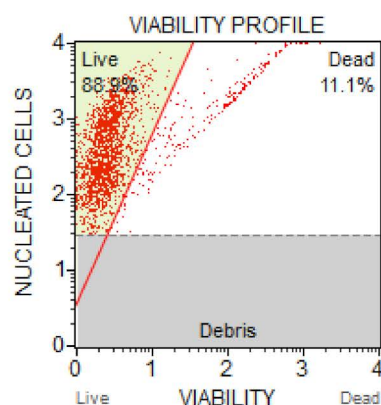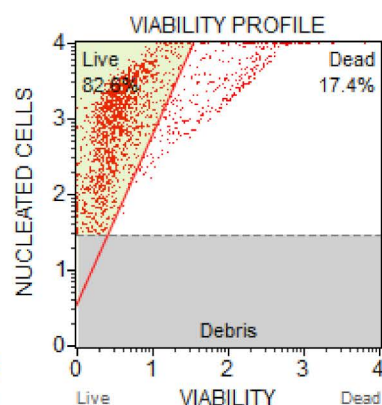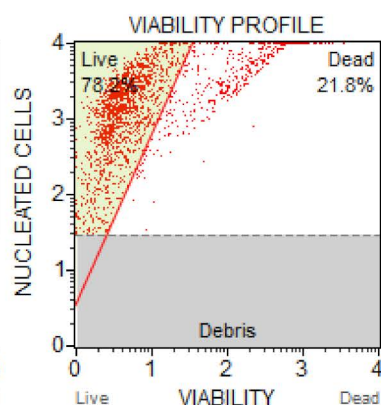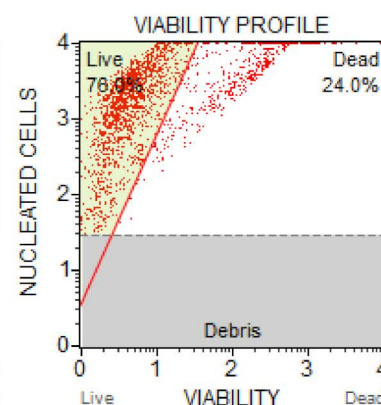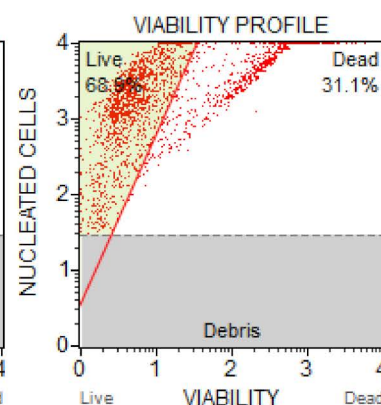

0923-R132H  
-dox

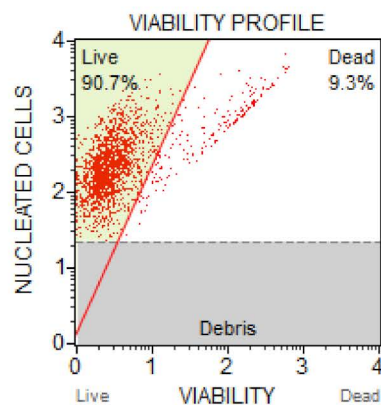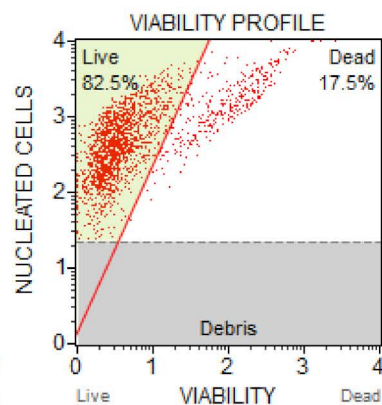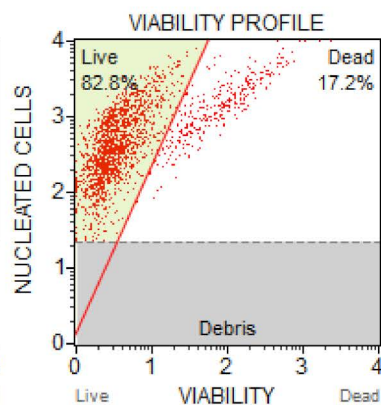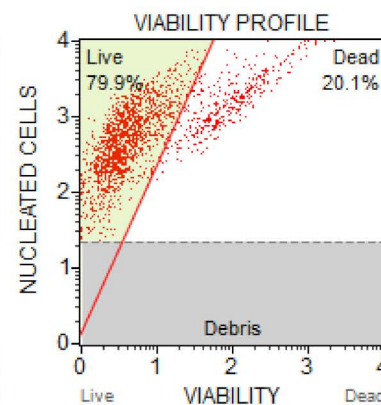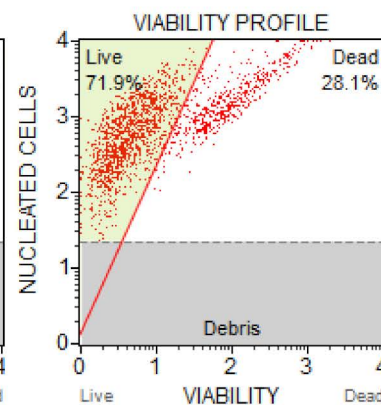

0923-R132H  
+dox

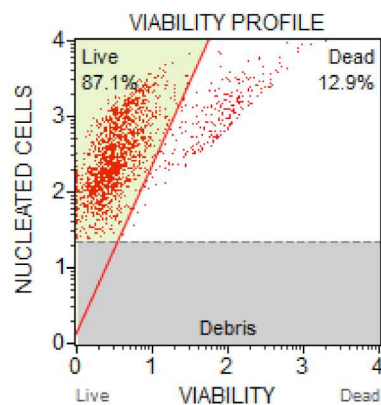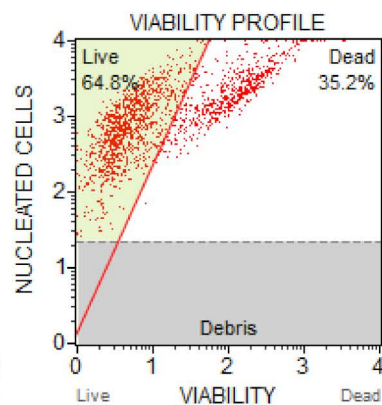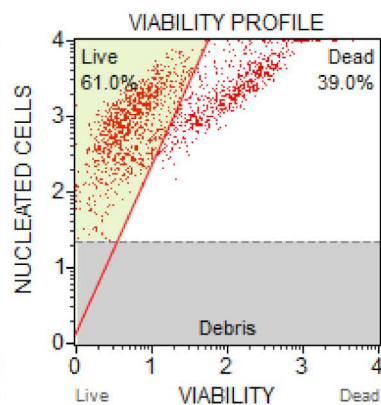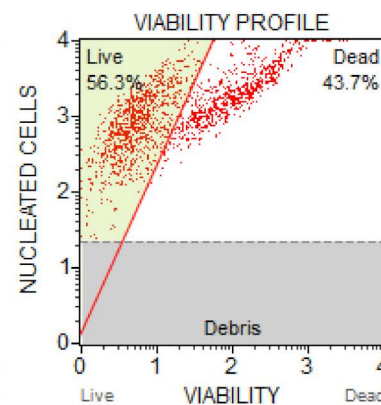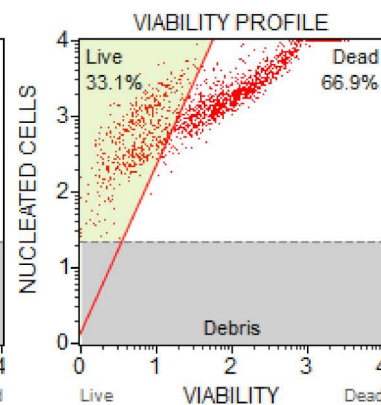

Supplement: Supplementary file 3 — Supplementary file3 (PDF 778 KB) [file 432_2022_4018_MOESM3_ESM.pdf]
